# Is My Stress Out of Place? Bread Wheat Response to Saline Stress Varies in Pattern and Extent Across Experimental Settings

**DOI:** 10.1002/pld3.70088

**Published:** 2025-07-02

**Authors:** Anne Plessis

**Affiliations:** ^1^ School of Biological and Marine Sciences University of Plymouth, Drake Circus Plymouth UK

**Keywords:** controlled conditions, ecophysiology, experimental conditions, field, greenhouse, salt stress, *Triticum aestivum*

## Abstract

Adopting crops and agricultural practices that help sustain yield under abiotic stress will be a major element of future food security under climate change. However, little of the intensive research into the mechanisms of plant abiotic stress response has translated into improved yield stability. A suspected obstacle to translatability of research findings in this area is artificial experimental conditions, but we lack evidence to support this explanation. Here, we combined a meta‐analysis and an experimental approach to compare the effect of salt stress on wheat yield, growth, and physiology across four distinct experimental settings: field/field‐like conditions, potted plants in a climate chamber, in a greenhouse, and outdoors. The meta‐analysis, comparing responses relative to control conditions over similar ranges of salt stress intensity, confirmed that field conditions led to more limited impact on yield than in the other three experimental settings and uncovered differences in how shoot and root biomass are relatively affected by salt stress between greenhouse and outdoors pot experiments. In our experiment, we identified very distinct responses for each of the four experimental settings, with plants outdoors accumulating more Na^+^ and proline than plants indoors, and shoot growth and yield were least affected by stress in field‐like conditions and most affected in the climate chambers. Together, these results suggest that the nature of the acclimation mechanisms used by wheat to face salt stress can depend on the experimental setting. While our findings need confirmation for other crops and abiotic stresses, we recommend renewed attention to the conditions under which experiments are carried out and to favor more realistic growth conditions when possible.

**Abbreviations**
CC: climate chamber
EC: electrical conductivity
GH: greenhouse
OP: outdoors pots
TKW: thousand kernel weight

## 1 Introduction

To mitigate the yield loss linked to abiotic stress that will escalate with climate change, new agricultural practices and tolerant crops need to be developed (Hopmans et al. 2021; Benitez‐Alfonso et al. 2023). A plethora of studies has investigated plant molecular responses to abiotic stress in order to identify genes that can be modified or introduced to improve crop resilience to adverse climatic and soil conditions (Mickelbart et al. 2015; Bowerman et al. 2023; Sato et al. 2024). With the development of CRISPR‐CAS9 gene editing (Manghwar et al. 2019), there is hope that this mechanistic knowledge can be swiftly translated into a range of climate‐smart crops (Liu et al. 2023). Despite all this work, attempts to improve crops for climate resilience using genetic engineering have not met expectations so far (Araus et al. 2019; Chan et al. 2020; Passioura 2020). Only a handful of genetically engineered crops with improved yield under drought (none for salt or heat stress) are on the market or have been approved for commercialization (Sato et al. 2024). Breeding for yield stability under abiotic stress has proved more successful, especially for maize (Messina et al. 2023) and rice (Hu and Schmidhalter 2023), but drought and soil salinity resilience have proved more elusive in wheat and barley (Asif et al. 2019; Langridge and Reynolds 2021). To mitigate the impact of low‐quality soils and climate change on food security, we therefore need to solve the issue of the poor translatability of the research on crop abiotic stress resilience.

Some of the difficulties in improving crop yield stability under abiotic stress have been blamed on faulty experimental designs where abiotic stress treatments have little resemblance (e.g., in duration and intensity) to natural scenarios and are applied at a different stage than when they occur in the field (Passioura 2010, 2020; Claeys and Inzé 2013; Cabello et al. 2014; Plessis 2023; Vadez et al. 2024). The measurement of stress resilience as survival rather than yield under stress and genetic modifications being introduced in “old” genotypes rather than current elite cultivars also accounts for low translatability of research (Cominelli et al. 2013; Khaipho‐Burch et al. 2023). Another major shortcoming of abiotic stress experiments is the use of artificial growth conditions (greenhouse [GH] or controlled climate chambers [CCs]), which is believed to hinder the application of mechanistic knowledge or trait selection into field systems (Roy et al. 2014; Chan et al. 2020; Langridge and Reynolds 2021; Plessis 2023).

There have been many observations of discrepancies between studies conducted in CCs, GHs, and the field, with genotypic effects varying widely across experimental settings. For example, Sales et al. (2022) found no correlation between field and GH measurements of photosynthesis across 80 wheat cultivars. When Brachi et al. (2010) mapped QTLs for Arabidopsis flowering time in the field, most of the genes and genomic regions they identified did not match those associated with flowering traits in previous GH studies. This interaction between environmental conditions and genotype is also evidenced by Wilczek et al. (2009), who found that Arabidopsis plants holding mutations affecting flowering time under controlled conditions generally flowered at the same time as the wild type in the field. Mismatches between controlled and field conditions are also found at the molecular and metabolic levels. Maize genes annotated as involved in abiotic stress response showed much higher expression in the field than in a growth chamber (Nelissen et al. 2020), and a similar result was obtained in rice, even though the growth chamber simulated outdoor fluctuations in irradiance, temperature, and humidity (Hashida et al. 2022). Arabidopsis plants grown in a chamber under controlled conditions displayed significantly different metabolic patterns to those grown in a GH (Annunziata et al. 2017). Given that the effect of abiotic stress and the success of traits for abiotic stress resilience are very dependent on the environmental context (Tardieu 2012; Hu and Schmidhalter 2023), it is not surprising that the few studies that have compared the performance of a range of cultivars under abiotic stress between different experimental settings have found inconsistent results. Telfer et al. (2018) identified negative correlations in two measurements of heat stress tolerance across 24 wheat cultivars between controlled and field environments. While nitrogen use efficiency–related traits were consistent between GH and field across six wheat cultivars subjected to drought and varying levels of fertilization, there was no correlation between these two experimental settings for biomass and yield (Asplund et al. 2014).

These discrepancies can be linked to the many differences in environmental conditions between artificial growth conditions and field conditions that are likely to affect plant growth and physiology. On average, in controlled conditions, the temperature is higher and the light intensity lower than in the field, which leads, among other things, to elevated growth rates (Poorter, Fiorani, et al. 2016). Growing plants in pots constrains root development, affects root temperature, modifies water and nutrient uptake, and can generate hypoxic conditions damaging to the root system (Passioura 2006; Poorter, Bühler, et al. 2012). Pot growth will also typically result in lower plant density than in agricultural fields, especially for cereals, leading to reduced competition for light and, in turn, higher biomass and distinct plant architecture (Postma et al. 2021). In controlled conditions, plant watering is usually done in such a way that little water lands on the leaves, while outdoors leaf wetting is a frequent occurrence that can impact plant physiology, in particular water relationships (Dawson and Goldsmith 2018). Wind is also an overlooked aspect of natural environments; yet, it has a major impact on plant morphology and internal structures (Gardiner et al. 2016) and its absence in controlled conditions is bound to lead to altered growth patterns, that is, taller plants with thinner stems. Finally, other conditions being comparable, plants grown under fluctuating lights tend to have lower biomass, higher leaf specific area, and different photosynthetic pigment composition than those grown under stable light (Morales and Kaiser 2020).

As a result of these fundamental differences between experimental settings, there is a significant risk that the mechanisms identified as contributing to stress resilience under controlled conditions will differ from those at play in field conditions. Yet, we know little about how experimental settings as a whole affect developmental and physiological responses to abiotic stress or which aspects of artificial conditions have the most influence on stress responses. In this study, we used bread wheat ( *Triticum aestivum* ) under salt stress as a case study to decipher and quantify the effects of the main “unrealistic” aspects of controlled conditions on plant stress physiology. To compare the effect of salt stress between field or field‐like conditions, potted plants outdoors, in GH and CC conditions on a range of growth and physiological measurements, we combined an experiment where the same salt stress protocol was applied across those four experimental settings and a meta‐analysis of single experimental setting salt stress studies.

## 2 Methods

### 2.1 Meta‐Analysis

#### 2.1.1 Study Search and Screening

In our meta‐analysis, we included data from studies selected on five criteria: (1) bread wheat ( *Triticum aestivum* ) was included; (2) plants were grown and subjected to varying levels of salinity in the growing medium, measured as electrical conductivity (EC), molar concentration, or ppm, including a “control” low salinity treatment, and the salt treatment was not highly sodic, alkaline, or with some other form of toxicity (e.g., boron), so that changes in plant growth or physiology were due entirely to the intensity of the salt stress; (3) roots were supported by soil, sand, vermiculite, perlite, cocopeat, or a mix of those; (4) experimental setting was provided and fit within one of four broad categories: field, outdoor pots (including net houses, wire houses and under rainout shelters, or some form of roof to exclude precipitations), pots in the GH, and pots in climate‐controlled growth chambers, and plants were grown in a single of these experimental settings; (5) yield, growth, physiological, or biochemical measurements were included.

We chose search terms to include as many relevant papers as possible mentioning our species and stress of interest; to avoid studies focusing solely on other aspects than the ones we chose to focus on (e.g., only gene expression), we also added as search terms two measurements we expected to be included in studies either focusing on productivity (“yield”) or metabolic aspects of salt stress response (“proline”). Therefore, we searched in Web of Science using the following search prompt “(wheat OR *Triticum aestivum* ) AND (salt OR salinity OR saline) AND (yield OR proline),” with an “All fields” search and excluding reviews, and in Scopus using the same search prompt, with an “Article title, Abstract, Keywords” search. We excluded review articles from both searches. On June 11, 2024, these searches provided 5022 studies from Web of Science and 1424 from Scopus. The search results were imported into Rayyan, an online tool for screening scientific articles (Ouzzani et al. 2016), where 1119 duplicate studies were manually removed and a first screening step was performed based on title and abstract to exclude studies that did not match our criteria. After this first screening step, 1150 studies were kept. A second screening step was then carried out based on the full text of the studies, to exclude more studies that did not fit our meta‐analysis criteria. We checked that the study included bread wheat (when the species was not provided, but the cultivar was mentioned, we searched the literature to find whether it was a *Triticum aestivum* cultivar), and we excluded 24 studies where the control was not comparable enough to the stress conditions, in particular when the control field was located too far away (over 50 km) from the saline field.

A pilot screen was conducted to select variables related to salt stress response that would be commonly measured across the different experimental settings. We listed the variables measured in 58 studies that fit the criteria for our meta‐analysis and recorded the experimental setting. We found that some measurements were very rarely measured outside of the field (e.g., days to anthesis), while others often measured in pot experiments were generally not measured in the field (e.g., root length or quantifications of ROS scavenging enzymes); finally, some measurements were not measured often enough generally (less than 20% of studies) that we would expect enough data to detect differences between experimental settings (e.g., Fv/Fm, grain protein content, and photosynthetic rate). Consequently, we restricted our search to papers including the following measurements that were commonly included across several experimental settings in our pilot: plant height, shoot dry weight, root dry weight, grain yield, thousand kernel weight, number of spikes, number of grains, chlorophyll, leaf or shoot proline, and Na^+^.

#### 2.1.2 Data Collection and Processing

We collected data presented in the articles as averages over replicates in tables and figures. When several genotypes of bread wheat were used or when experiments were repeated across years without a change in protocol (e.g., timing and intensity of salt stress), data were also averaged across genotypes and/or years. We did not average across treatments applied in combination to salt stress unless averages across treatments were provided for each level of salt stress in a more accessible and precise way (i.e., table vs. figure) than for individual treatments. We only collected data where the salinity induced a decrease in either shoot dry weight or yield.

For the quantification of Na^+^, proline, and chlorophyll in leaves/shoots, we only recorded data presented as amount per dry weight of tissue; this is because salt stress can lead to changes in total water content; therefore, differences in concentration per fresh weight can confound the effect of water loss (or gain) with the accumulation/degradation of the compound quantified. Some of the variables in our selected set were sometimes calculated from one or several other variables when they were not presented in the study (e.g., concentration in dry weight from concentration in fresh weight using total water content, itself calculated from shoot fresh weight and dry weight, or number of grains per plant from grain yield per plant and thousand kernel weight). For each growth‐related variable (height, shoot dry weight and root dry weight), we created two subvariables: one for measurements at the vegetative stage (before booting) and one for measurements at booting or later. Some variables included different methods of measurements, for example, chlorophyll measured through biochemical quantification or SPAD and yield measured per plant or per surface area.

Several aspects of the study methodology were recorded: experimental setting, level of stress, and timing of stress treatment. All measurements of salinity in the growing medium or irrigation solution were converted to EC using the following formulae: 1‐M NaCl is equivalent to 98 dS m^−1^ and 1000‐ppm NaCl is equivalent to 1.66 dS m^−1^. A relative EC was calculated as follows: salinity stress EC − control EC, in order to have comparable measurements of stress salinity between studies where the salinity of the soil was directly provided and those where only the salinity of the solution applied to the soil was given (usually no baseline salinity for the control treatment was provided); this way the salinity of the control treatment was artificially set to zero for all studies. When available, we also recorded the start and end point of the saline stress (either as days or growth stage); unless it was specified that the salt was flushed from the medium, we considered that once the stress was applied it lasted until the final measurement.

We screened our dataset for potentially duplicated data to eliminate duplicates missed during the initial screen, similar datasets published across different articles or studies that shared part of their datasets (20 studies in total). Two studies with obvious mistakes, for example, measurements orders of magnitude out of the range found in other studies, were removed. The final dataset that was analyzed came from a total of 279 articles (Table S1) that fit our criteria and where we were able to collect the necessary information for the data to be analyzed; this dataset contained a total of 539 unique salt stress observations (which includes observations from the same experiment but with different levels of stress), 117 from field experiments, 77 from outdoors pot (OP) experiments, 296 from pots in a GH, and 49 from pots in a CC, and 284 associated control observations (this is higher than the number of articles, as some of those include several experiments with their own control).

#### 2.1.3 Data Analysis

The data analysis was carried out in R (R Core Team 2021), and plots were created using the sciplot package (Morales and Murdoch 2020). To reduce variability between studies, stress response was calculated as the fraction of the stress value to the control value, that is, the relative value (Poorter, Niinemets, et al. 2010). Our analysis was based on the assumption that the effect of the salt stress increased with the intensity of the stress (Steppuhn and Wall 1997), so response was generally related to the level of salinity. A preliminary analysis showed clear correlations between most variables and EC of the stress treatment. However, for growth measurements taken at the vegetative stage, we found that the duration of the stress treatment impacted the extent of the response and that there was a better correlation between response and an alternative measurement of stress obtained by multiplying EC and duration of the treatment (thereafter called “stress integral”) than with EC alone.

To allow for statistical comparison between experimental settings across a dose–response curve, we chose to group data points within bins covering parts of the range of relative EC values or stress integral. The number and range of bins were adjusted for each variable, in order to avoid having fewer than five data points per setting in each bin and to have the most comparable average EC across settings for each bin. Outliers are a major issue in meta‐analyses and can significantly skew their outcome (Hunter and Schmidt 2004). We used a Hampel filter (Pearson et al. 2016), in which, within a given bin for a given setting, data points beyond three median absolute deviations of the median of the bin were excluded from the analysis.

Differences between each pair of experimental settings were tested for the data included across the overlapping bins for that pair. Normality of untransformed and log‐transformed data was determined using the Shapiro Wilk test, and variance homogeneity was tested with the RVAideMemoire R package (Herve 2025). If variances were equal and untransformed or log‐transformed data were normally distributed, we used the permutation‐based t‐test from the RVAideMemoire R package (Herve 2025) on untransformed or log‐transformed data, as relevant, to test the difference between the pair of experimental settings. If neither the untransformed nor log‐transformed data were normal, or if the data failed the variance homogeneity test, we used a Mann–Whitney *U* test instead.

### 2.2 Experimental Work

#### 2.2.1 Plant Material and Growing Conditions

All experiments were conducted on spring wheat ( *Triticum aestivum* ) cv. Tybalt (Limagrain, Saint Beauzire, France, https://www.limagrain.com/). Seeds were sown in wet compost in individual wells of a germination tray in a GH and transplanted to the different experimental settings a week after sowing.

The GH pots, OPs, and raised bed (RB) experiments took place from February to July 2022 in the experimental “Skardon” garden at the University of Plymouth, Plymouth, United Kingdom (50°37′ N 4°14′ W), all within 15 m of each other. The four RBs and OP experiment were located next to each other (with the pots equally split in four groups at the edge of each RB; Figure S1) under a rain out shelter in the form of a polytunnel that could be opened and closed manually. The shelter was open for the majority of the experiment, so the plants experienced natural outdoors conditions; it was closed shortly before heavy precipitation to avoid rainwater draining the salt from the pots or too deep in the soil of the RBs. Caution was taken for the shelter to always be open under sunny weather to avoid creating a GH effect artificially increasing temperature. The CC experiment was conducted in a Sanyo growth chamber (photosynthetically active radiation 183 μmol m^−2^ s^−1^) after the other experiments were completed. Photoperiod and day and night temperatures were adjusted weekly to match the average of the conditions in the GH experiment (Table S2).

For CC, GH, and OP, three seedlings were transplanted in 3‐L black plastic square pots (15 cm × 15 cm at the top and 20 cm high) containing 3 kg of a mix of 50% Devon farming soil, 40% soil from the experimental garden, and 10% compost (Peat Free Multi‐purpose with added John Innes, Westland Horticulture Ltd., Dugannon, Northern Ireland, UK). Mixing of the different components of the growth medium was carried out in the top 50 cm of the RBs, on top of the natural garden soil, and pots were filled with that same medium. Pots were saturated with water and left to drain to establish full pot water capacity. For the RB experiment, seedlings were transplanted at a density of 220 plants m^−2^, which is within the range of density for wheat in the United Kingdom.

Pots and RBs were manually weeded as necessary. Fertilization was provided in the form of a 0.75% dilution of Wilko lawn feed (15‐3‐3 NPK with iron), two doses of 50 mL per pot or 2 L per RB (equivalent volume per surface) during the vegetative stage.

#### 2.2.2 Stress Treatment

In all experiments, the salt stress treatment started at the two tiller‐stage, approximately a month after sowing, with the exact time varying slightly between experiments (between 28 and 36 days after sowing), as seedlings grew faster in GH and CC. Salt stress was applied using a solution of sea salts (Instant Ocean, Blacksburg, VA, USA, https://www.instantocean.com/; Table S3) that represents better the mix of ions found in brackish water and saline soils than pure NaCl, dissolved in rain water for RB, OP, and GH, and in distilled water for CC; rain water and distilled water were also used for control watering. Each pot was watered with 50 mL of salt solution and each RB with 2 L of salt solution, which amounted to the same volume per surface three times a week. The concentration of salt was gradually increased by 2 g L^−1^ increments over 3 weeks until the final concentration of 22 g L^−1^ (27 dS m^−1^) was reached. This was done to mimic the slow increase in soil solution salinity that takes place during the growing season in saline agricultural fields and avoid osmotic shock (Shavrukov 2013). Drainage of soil solution was limited to a minimum, and the pots were never flushed with nonsaline water. The salt stress treatment was maintained until there was no more green leaf tissue visible in stressed plants, which amounted to 92 days of treatment for GH, 94 days for CC and OP, and 110 days for RB. Soil water content was maintained at 70% pot water capacity in both control and stressed pots from transplanting until all plants in a pot had no more green tissue visible. Comparable soil moisture was obtained in the RBs as in the pots using a moisture meter (HH2 ThetaProbe, Delta‐T Devices Ltd, Cambridge, UK, www.delta‐t.co.uk).

Soil salinity was measured at the end of the experiment at different depths for the pots that had been watered with the saline solution using the EC1:5 method (He et al. 2012). For pots, soil samples were taken from the first two top cm, middle of the pot, and last 2 cm at the bottom of the pot; for the RBs, soil samples were taken from the first two top cm, 4–9 cm deep, 10–14 cm deep, and 23–28 cm deep in at least two replicates for each experimental setting. The baseline EC1:5 value for the soil used in the experiment was 0.35 dS m^−1^. Similar results were obtained for the pots in CC, GH, and OP settings, with EC1:5 values around 21 dS m^−1^ for the top 2 cm of soil, 3.5 dS m^−1^ at middle depth, and 7.5 dS m^−1^ at the bottom of the pot. For the RBs, the top 2 cm had an EC1:5 of 6.8 dS m^−1^, 2.4 dS m^−1^ at 4–9 cm deep, 1.4 dS m^−1^ at 10–14 cm deep, and 1.2 dS m^−1^ at 23–28 cm deep.

#### 2.2.3 Plant Sampling, Growth, and Yield Measurements

Plant height (from the soil to the end of the longest leaf) was recorded weekly from the start of the stress treatment for six randomly selected plants per treatment in GH and CC, for eight plants in OP and 16 plants in RB. More plants were measured for OP and RB to account for the added variability created by the split into two blocks per treatment and the highest variability observed for plant size in RB.

Four plants per experimental setting and treatment were harvested at three different times during the experiment: after 23–25 days of stress (tillering stage for CC, OP, and RB, stem elongation stage for GH), after 29–31 days of stress (tillering stage for CC, end of tillering stage for OP and RB, stem elongation stage for GH) and after 46–49 days of stress (tillering stage for CC, start of booting stage for OP and RB, end of booting stage for GH). Whole shoots were harvested and rinsed to remove salt from stress treatment watering, with the ears removed when they were present, and the shoots without ears were frozen in liquid nitrogen. Sampling was always carried out around 9 a.m. to avoid circadian variations in metabolites between sampling points. For CC, GH, and OP, only one plant per pot was harvested over the three sampling times for each pot, so at maturity, there were two plants in each pot.

Harvesting was done once plants had reached maturity. For CC, GH, and OP, all remaining plants (18 per treatment, minus six for OP control and four for OP salt because of an undiagnosed disease that affected yield); for RB, 30 plants (none from the edge of the plot and none with the same undiagnosed disease as some OP plants) per treatment were randomly harvested. Plants were dried in an oven overnight at 60°C to evaporate any remaining humidity. Shoot biomass, including generative organs, was recorded, and for each plant, spikes and grains were counted and weighed.

#### 2.2.4 Shoot Tissue Analyses

Whole shoots were freeze dried for at least 48 h, ground into a fine powder using a coffee grinder, and stored in dry conditions.

Sodium and potassium were quantified using flame photometry. The method was adapted from (Munns, Wallace, et al. 2010). About 15 mg of tissue powder was digested in 2 mL of 0.5‐M nitric acid for 1 h at 80°C. After 10 min of centrifugation, the supernatant was collected and diluted 5 times (control samples only for sodium analysis) and 50 times. Diluted samples and standards were quantified using a flame photometer (Model 420, Sherwood, Cambridge, UK, sherwood‐scientific.com).

Proline quantification was adapted from Shabnam et al. (2016). Twelve to 15 mg of tissue powder were extracted with 40% ethanol containing 10‐mM ascorbate for 18 h at 4°C. After 10 min of centrifugation, 33 μL of the supernatant was mixed with 66 μL of 1.25% w/v ninhydrin in glacial acetic acid in 200 μL PCR tubes and incubated at 100°C for 30 min. This was replicated for each sample, replacing the ninhydrin reagent with glacial acetic acid as a “sample blank” to assess the baseline coloration (independent of the ninhydrin reacting with proline) of each sample. The content of each tube was transferred to a 96‐well plate and absorbance at 520 nm was measured in a plate reader (Spectramax Plus 384, Molecular Devices, San Jose, CA, USA, moleculardevices.com/). Proline concentration was calculated using the values for standards included in each set of reactions and by subtracting the absorbance value for the “sample blank.”

Chlorophyll extraction was modified from Sims and Gamon (2002). About 12 mg of dry leaf powder was extracted twice with 1.8 mL of 80:20 (v/v) acetone Tris buffer (pH 7.8), shaking for 5 min, followed by 5 min of centrifugation. Absorbance was measured at 537, 647, and 663 nm, and chlorophyll concentration was calculated using the equations provided by Sims and Gamon (2002).

#### 2.2.5 Data Analysis

Data analysis was carried out in R (R Core Team 2021), and some of the plots were created using the sciplot package. The interaction between salt stress treatment and experimental setting were analyzed using a two‐way ANOVA. When the residuals of the ANOVA were determined as not normally distributed by a Shapiro–Wilk test, it was replaced with a nonparametric two‐way Scheirer–Ray–Hare test.

## 3 Results

### 3.1 Yield Components and Biomass Accumulation

#### 3.1.1 Meta‐Analysis

**FIGURE 1:**
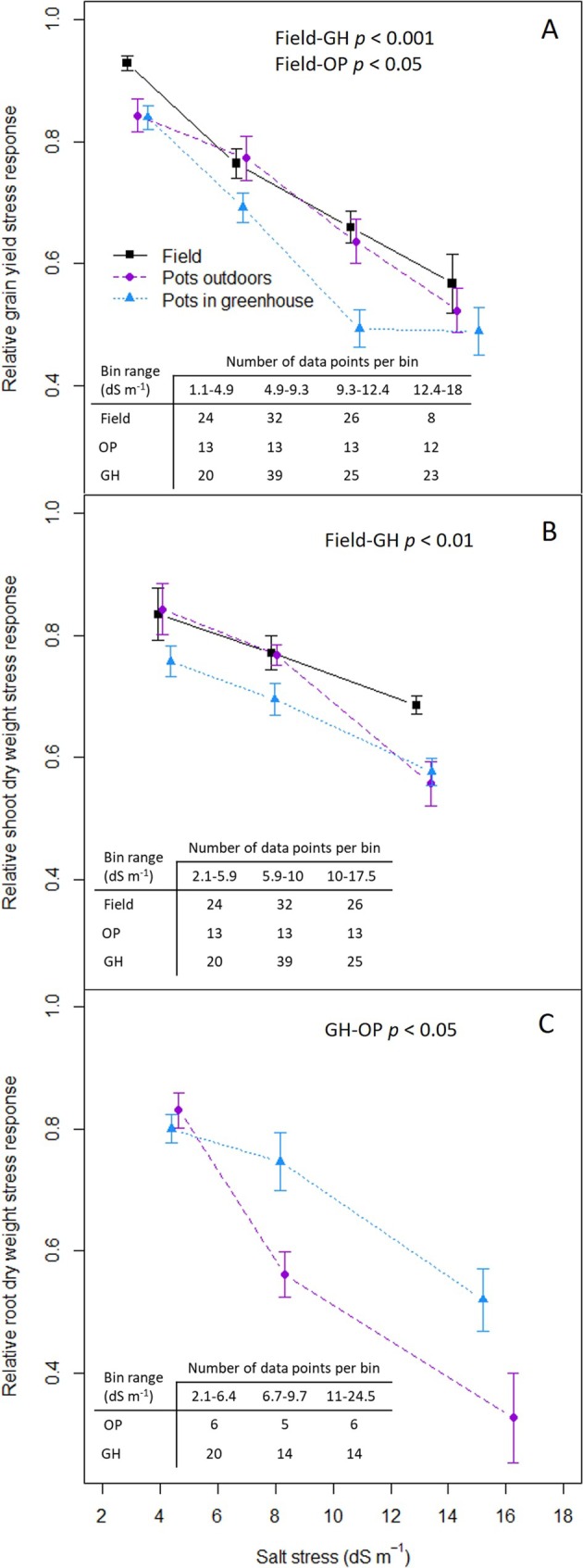
Meta‐analysis of salt stress response of yield and growth in three experimental settings and across different levels of salinity. Ratio of grain yield (per plant or per hectare) in saline stress conditions to control conditions (A). Ratio of shoot dry weight in saline stress conditions to control conditions (B). Ratio of root dry weight in saline stress conditions to control conditions (C). Field: black squares; pots outdoors: purple circles; pots in a greenhouse: blue triangles. Number of data points in each bin of salinity presented in the tables within each plot; statistical analysis for pair‐wise comparison of settings included at the top of each plot, obtained with Mann–Whitney *U* test for A and B, permutation *t* test for C; error bars represent the standard error.

The decrease in response to salt stress is significantly higher for yield and yield components in GH pot experiments than in the field (Figures 1 and S2). The yield response to salt stress in OP experiments is significantly different from the field and falls in between the field and the GH for most levels of stress (Figure 1A,B). A similar pattern of differences between experimental settings is observed for shoot dry weight, which is considered a good proxy for yield, with a significantly stronger negative response in the GH pots than in the field (Figure 1B). In contrast to yield and shoot dry weight, root dry weight is significantly more affected by salt stress at higher levels of EC in OP experiments than in GH conditions (Figure 1C). No data were available for root dry weight in field conditions to assess whether the similarity to OP in yield response to salinity holds true at the root level.

**FIGURE 2:**
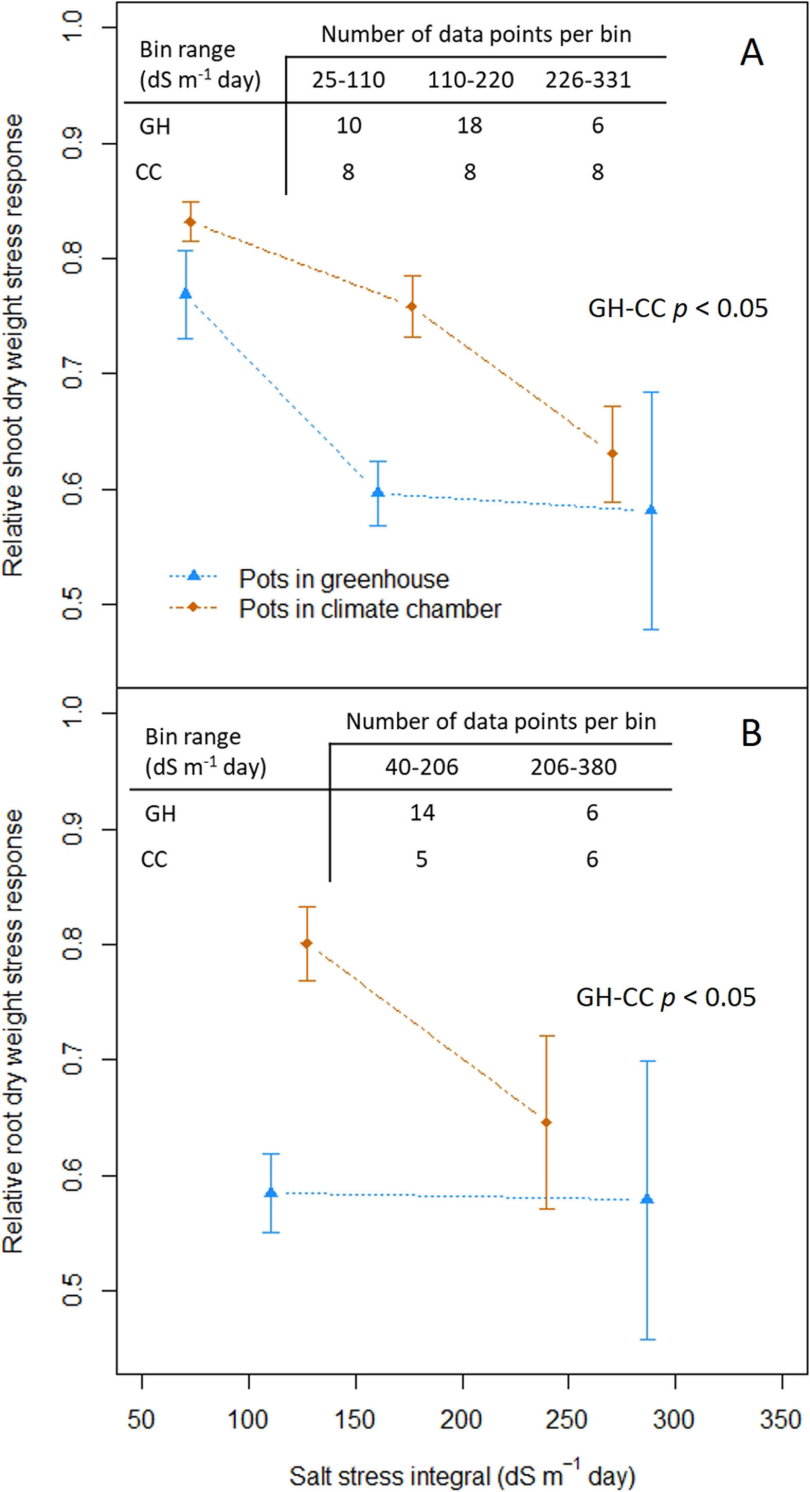
Meta‐analysis of salt stress response of growth measured at the vegetative stage in two experimental settings and across different levels of salinity. Ratio of shoot dry weight in saline stress conditions to control conditions (A). Ratio of root dry weight in saline stress conditions to control conditions (B). Pots in a greenhouse: blue triangles; pots in a climate chamber: orange diamonds. Number of data points in each bin of salinity presented in the tables within each plot; statistical analysis for comparison of settings included in the middle of each plot, obtained with a permutation *t* test; error bars represent the standard error.

Very little data for the reproductive stage and maturity are available for CC experiments; therefore, we were limited to the vegetative stage to compare growth variables from CCs to other experimental settings, and comparison was only possible with GH experiments as there are not enough vegetative stage data collected from field and outdoor pot studies. For vegetative stage growth measurements, as described in the methods, an integral of salt treatment EC over the stress duration was used to represent stress level. The negative effect of salt stress is significantly higher in the GH than in the CC for both shoot and root dry weight (Figure 2).

#### 3.1.2 Experimental Study

**FIGURE 3:**
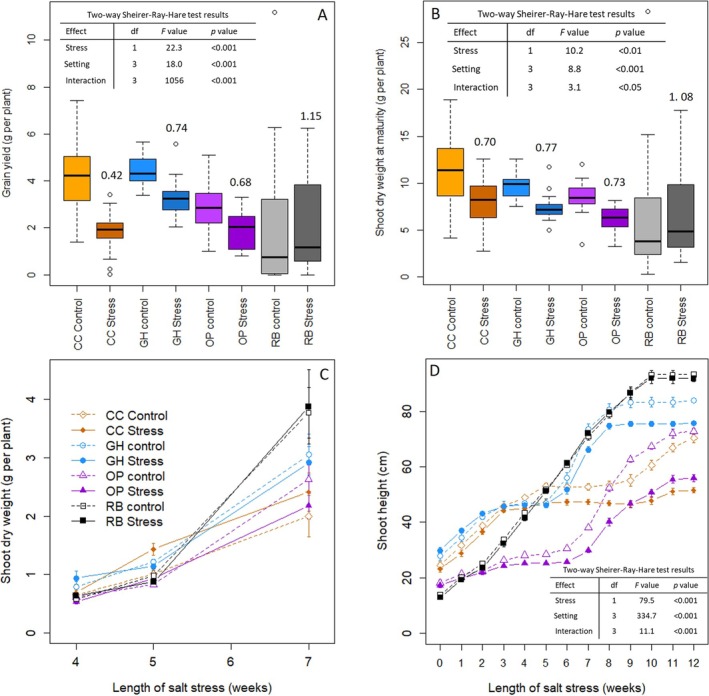
Yield and growth for wheat subjected to salt stress in four experimental settings. Weight of grain yield per plant (A). Shoot dry weight at maturity (B). For A and B median represented by black line and mean by dark dot, the box represents the first and third quartile of the data and the whiskers the range of the data, excluding outliers; the number on top of stress boxes is the relative value of the variable (ratio of stress to control values); statistical analysis included at the top of each plot. Accumulation of shoot biomass during 7 weeks of stress; bars represent the standard error for *n* = 4 (C). Increase in shoot height over 7 weeks of stress; bars represent the standard error for *n* = 6 for CC and GH, *n* = 8 for OP and *n* = 16 for RB and statistical analysis for the final point of the time series presented in the table at the bottom of the plot (D). CC = climate chamber, orange, GH = greenhouse, blue, OP = outdoors pots, purple and RB = outdoors raised beds, gray; control: light shade (A and B) or open symbol (C and D); saline stress: dark shade (A and B) or filled symbol (C and D).

The negative effect of salt stress on yield was strongest in the CC, followed by outdoor pots (Figure 3A). While there was no deleterious effect of salt stress on yield in the RBs, the stress did lower TKW in a comparable manner to results in the GH and OP (Figures 3A and S3A). In contrast to the results of our meta‐analysis, in our experiment, there was little, if any, negative effect of salt stress on the number of grains and spikes per plant across all our experimental settings (Figures S2B,C and S3B,C). The effect of salt stress on shoot dry weight across the different experimental settings was mostly in line with what we observed for yield, but with the effect of the stress more limited, especially for plants in the GC (Figure 3B). We noted that, independently of the stress, the time pattern of growth of the wheat plants was very variable across the experimental settings, with plants growing more slowly during the vegetative stage in the OPs, while plants in the GH reached their final height earlier than in the other experimental settings (Figure 3C,D).

### 3.2 Cellular Damages and Acclimations

Saline stress gives rise to damages linked to osmotic and ionic stresses, with one such damage being the degradation of chlorophyll due to oxidative stress in the chloroplast. Acclimations are used to mitigate those damages, in particular osmotic adjustment, which decreases the plant osmotic potential to avoid cellular desiccation (Sanders and Arndt 2012). Both proline and Na^+^ accumulation contribute to osmotic adjustment, but high levels of Na^+^ can be deleterious to the plant (Munns, James, et al. 2016).

#### 3.2.1 Meta‐Analysis

**FIGURE 4:**
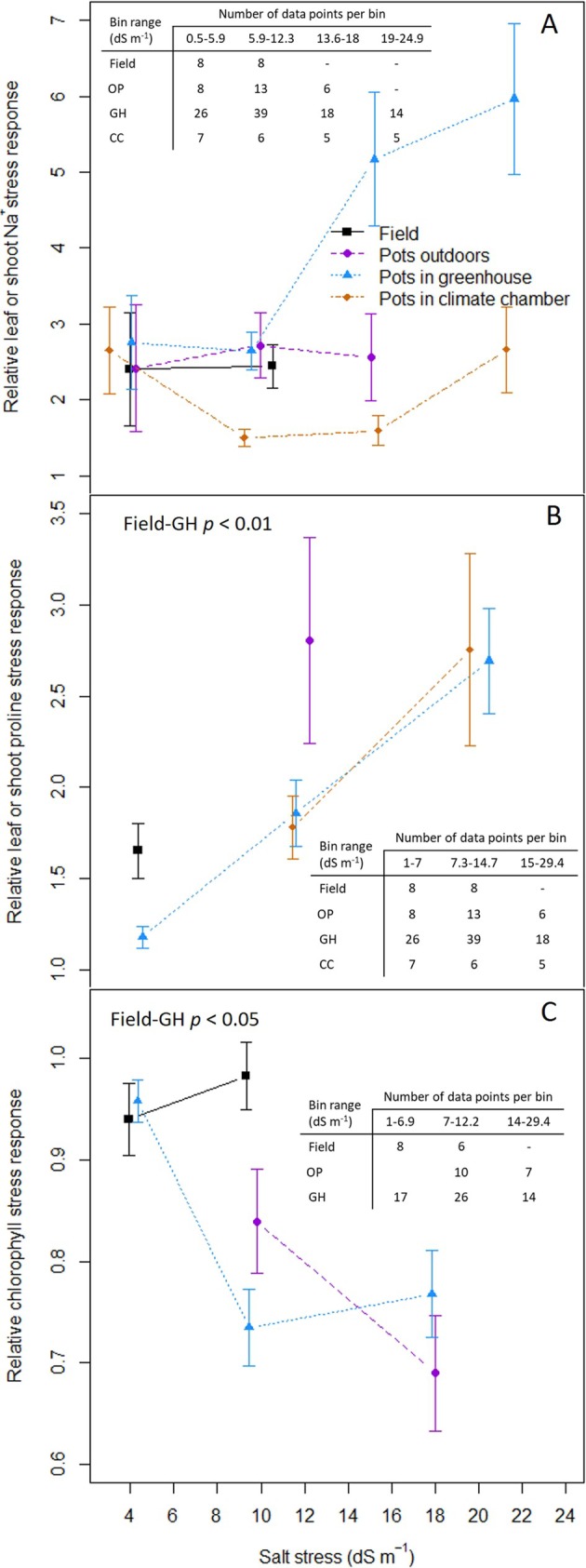
Meta‐analysis of cellular responses to salt stress in four experimental settings and across different levels of salinity. Ratio of shoot or leaf Na in saline stress conditions to control conditions (A). Ratio of shoot or leaf proline in saline stress conditions to control conditions (B). Ratio of chlorophyll concentration in saline stress conditions to control conditions (C). Field: black squares; pots outdoors: purple circles; pots in a greenhouse: blue triangles; pots in a climate chamber: orange diamonds. Number of data points in each bin of salinity presented in the tables within each plot; statistical analysis for pair‐wise comparison of settings included at the top of each plot, obtained with Mann–Whitney *U* test for A (no significant difference) and C, permutation *t* test on log transformed data for B; error bars represent the standard error.

We observed higher accumulation of Na^+^ for pot‐grown plants in a GH than in a CC, but this difference was not statistically significant (Figure 4A). Because of limited data available for field and outdoor pot conditions, it is difficult to draw conclusions for these two experimental settings, but our results suggest that Na^+^ accumulation does not increase as much with salinity outdoors as in a GH (Figure 4A). In contrast, there was very little difference in proline accumulation between GH and CC (Figure 4B), but we did detect a significantly higher accumulation of proline in the field than for pots in a GH. In the case of chlorophyll, sufficient data to carry out the analysis was only available for limited ranges of stress intensities, yet our data indicate significantly lower levels of chlorophyll in the GH than in the field (Figure 4C), with notably little impact of salinity stress on chlorophyll levels at medium levels of stress intensity in the field.

#### 3.2.2 Experimental Study

**FIGURE 5:**
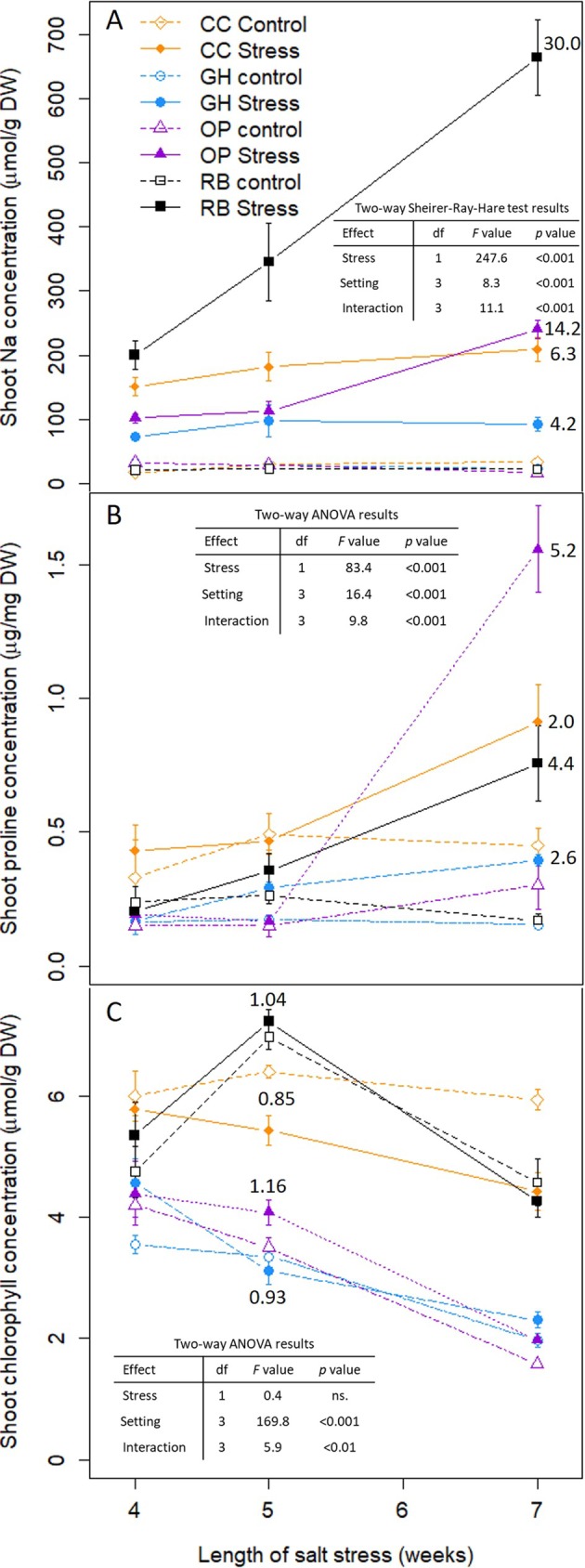
Cellular responses to salt stress for wheat subjected to salt stress in four experimental settings. Shoot proline concentration over 7 weeks of stress; bars represent the standard error for *n* = 4; (A). Shoot Na concentration over 7 weeks of stress; bars represent the standard error for *n* = 4; (B). Shoot chlorophyll concentration over 7 weeks of stress; bars represent the standard error for *n* = 4 except for CC stressed at 4 weeks and RB stressed at 7 weeks where *n* = 3; (C). CC = climate chamber, orange, GH = greenhouse, blue, OP = outdoors pots, purple and RB = outdoors raised beds, gray; control: open symbols; saline stress: filled symbols Statistical analysis (table within the plot) and relative value of the variable (ratio of stress to control values, written near the stress symbol) were calculated for each variable for the time point with the highest average value over all treatments.

We looked at how stress‐related metabolites accumulated over time across the different experimental settings. Under salt stress, Na^+^ levels were higher, relative to concentrations in the control treatment, in pots outdoors and in the field than in the GH and the CC (Figure 5A), which contrasts with our meta‐analysis results where the highest relative accumulation of Na^+^ was for the GH (Figure 4A). We found a similar pattern of higher relative concentrations of shoot proline for field and OP (Figure 5B), which in this case was in accordance with what our meta‐analysis suggested (Figure 4B). In the case of chlorophyll, we did not detect a significant effect of stress, while there was a significant effect of experimental setting, with higher chlorophyll concentration in the RBs and CC than in the GH and OPs (Figure 5C). The interaction between stress and experimental setting was significant, with higher chlorophyll concentration in stressed than control conditions at most time points in the RBs, OPs, and GH, in contrast with lower chlorophyll concentrations for the salt treatment in the CC. These results diverge from our meta‐analysis, where, beyond moderate levels of stress, chlorophyll concentrations were lower under stress in GH and OP settings than in the field (Figure 4C).

## 4 Discussion

### 4.1 Salt Stress Response Mechanisms Can Vary Across Experimental Settings

We observed in both our meta‐analysis and experiment that yield and shoot biomass of wheat in field or field‐like conditions were less affected by salinity stress than in outdoor and GH pot settings. Supporting these findings, a meta‐analysis of 10 studies on alfalfa also found a more deleterious effect of salt stress on shoot dry weight in the GH than in the field (Bansal and Yin 2021). Similarly, in parallel field and GH experiments, Thapa et al. (2017) recorded deleterious effects of salt stress on wheat yield, root, and shoot growth in the GH but only on height for field plants subjected to the same stress; they attributed this difference to the uniformity of the salinity in their GH pots compared to the field. This explanation does not hold in our experiment, where there was acute variation in soil salinity with pot depth. However, salt was distributed throughout a much higher volume of soil in the RBs, resulting in overall lower salinity levels, providing an alternative explanation for the modest effect of salt stress on the RB plants. In contrast to our experiment, our meta‐analysis showed less difference in shoot‐related variables between plants grown in pots placed outdoors and those planted in the field than between plants in pots in a GH and outdoors, suggesting that differences between GH and outdoor conditions may also be responsible for the stronger effect of salt stress in GHs. In particular, maximum temperatures tend to be higher in GHs than outdoors or in CCs due to the GH effect and, in GHs with little or no ventilation, limited heat dissipation through air flux. Generally, in wheat, heat worsens the effect of salt stress on yield, biomass accumulation, and chlorophyll content (Altaf et al. 2021; Aouz et al. 2023; Da Ros et al. 2023; Chattha et al. 2024), so higher temperatures in GHs than in the other settings could contribute to the more severe effect of salt stress. Finally, while in our RBs and generally in agricultural fields, wheat density reaches several hundred plants per square meter, in pot experiments, including ours, density is often lower in pots due to edge effects and pot spacing, even if density within a pot is similar to field levels, so plants in pot experiments tend to experience less competition for light than in the field. As planting density has extensive effects on plant biomass and architecture (Postma et al. 2021) and can interact with the effect of salt stress (García‐Caparros et al. 2025), it is another factor that could explain the difference between field (or field‐like) and pot conditions.

Our results also reveal that cellular and root responses to the stress were not always affected by the experimental setting in the same way as yield and shoot growth, which is even more worrisome than a difference in the extent of the effect of the salinity stress between experimental settings. In our meta‐analysis, we found that salt‐stressed plants in a GH are more impacted at the shoot level than in outdoor pots, but less impacted in terms of root biomass. In our experiment, the salt stress‐induced accumulation of proline in the shoot (relative to control conditions) was greater in the RBs than in the GH and CCs despite a more severe effect of salt stress on yield and shoot growth in those controlled settings; this aligns with our meta‐analysis results for proline, where field plants accumulated significantly more proline than in the GH. In our experiment, we also found that the stressed RB plants accumulated much more Na^+^ in their shoots than in any other settings. Taken together, these results suggest that salt‐stressed plants rely on different strategies to acclimate to salt stress across settings, with plants in outdoor pots investing more in root growth than in the GH and plants in field or field‐like conditions relying more on osmotic adjustment, especially in our experiment where Na^+^ would have also contributed to the overall accumulation of solutes. This has strong implications for the validity of experiments investigating the mechanisms and traits involved in salt stress resilience, as our results indicate that a different set of acclimations may be put in place in the field than in other experimental settings. Varying contributions of salt stress resilience mechanisms across experimental settings have already been observed in barley, where plants relied more heavily on salt exclusion than osmotic adjustment in soil‐based experiments relative to hydroponics experiments (Tavakkoli, Rengasamy, et al. 2010).

There are many possible explanations for the differences in the pattern of response to salt stress across experimental settings, and it is likely several factors are responsible for the discrepancies observed. The different distribution of soil salinity and lower plant density in pots than in field or field‐like conditions, as well as the often higher average temperature in GHs, probably contribute to varying salt stress acclimation strategies across experimental settings. The majority of the data in our meta‐analysis comes from low‐latitude countries (Table S1) where GHs often need constant strong ventilation to avoid overheating, which in combination with high temperatures would lead to higher transpiration than in other experimental settings and could be why in that dataset we observed the highest accumulation of Na^+^ in GHs. Another explanation for the varying response patterns between experimental settings could be differences in developmental pace, which were observed through distinct curves of increase of plant height over time for each experimental setting. In particular, the transition to the reproductive stage was the latest in the RBs, which could have given the plants in this setting more time to acclimate to the high salinity before anthesis and grain development, which are stress sensitive stages (Atta et al. 2023).

### 4.2 Challenges and Future Work in Understanding the Consequences of Experimental Settings on Abiotic Stress Studies

We investigated the impact of experimental setting on the effect of salt stress with an experiment and meta‐analysis, each with a specific set of limitations. In our experiment, plants of the same wheat cultivar were grown alongside in the RBs, pots outdoors, and in the GH within a few meters of each other, while the temperature and photoperiod in the CC and GH were similar. This allowed us to analyze the effect of experimental setting independently of other factors (genotypic or environmental); however, these results are specific to the genotype, salt stress applied, and environmental conditions of this experiment, limiting its generalisability. In our meta‐analysis, we averaged results obtained over many climates, facilities, growing conditions, stress scenarios, and genotypes, making the results more generalizable, but the variability between experiments generates noisy data and may introduce confounding factors. For example, experiments in CCs focus preferentially on the vegetative stage, while field experiments rarely included vegetative stage measurements, and it is also possible that some genotypes are preferentially used in certain experimental settings.

While both our meta‐analysis and experimental work provide evidence for a significant impact of experimental setting on the pattern of salinity stress response in wheat, and in both cases, the stress had the least effect on yield and growth in field or field‐like conditions, the two approaches also provide contrasting results, possibly because of the limitations detailed above. In particular, our meta‐analysis found that GH conditions tend to exacerbate the effect of salt stress in comparison to the other settings, while in our experimental work, the impact of salt stress on yield and growth was stronger in the CC and OPs than in the GH. The likely difference in ventilation of our GH and GHs at lower latitudes mentioned earlier, possibly resulting in differences in transpiration, could explain this discrepancy. Another important inconsistency between the two approaches is the accumulation of Na^+^ under field conditions: in our experiment, the salt‐stressed RB grown plants reached a 30‐fold increase in shoot Na^+^ compared to the control, while in our meta‐analysis, we found that this increase was generally lower than three‐fold. This is particularly intriguing given how little the shoot growth of our RB plants was affected by the salt stress. It could suggest that in the conditions of our RBs, the acclimation put in place by the plants was similar to that of halophytic plants, using a resource‐efficient form of osmotic adjustment (Hopmans et al. 2021).

In our experiment, the salt stress unexpectedly led to positive, but nonsignificant, effects on yield and shoot growth in the RBs. We suspect that this result was not due to the salt stress but to an unidentified pathogen that affected each of our four RB plots unequally. It was difficult to quantify the exact impact of this pathogen on yield, and while we aimed to exclude plants visibly infected from our measurements, it is possible that our salt stress plots were less affected by this pathogen, confounding the effect of high salinity. Without this pathogen, the difference in the effect of the stress between the RBs and the other experimental settings may have been smaller, but we believe this issue does not invalidate the observed difference in acclimation strategy (e.g., accumulation of Na^+^) between experimental settings.

In contrast, one major advantage of the meta‐analysis is that the effect of such confounding factors will generally be minimized by the removal of outliers and averaging over many data points. Yet, this benefit is only achieved when sufficient data can be collected. As different stress scenarios tend to be applied across experimental settings (e.g., vegetative stage and cellular measurements in CCs; yield and yield components in the field), we were not able to collect datasets spanning all four experimental settings and the full range of salt stress intensities. This limited the scope of our statistical analysis because unbalanced comparisons between experimental settings meant that we had to use less sensitive tests of difference between means.

Given these limitations, it will be important to confirm the results we obtained for more species, other abiotic stresses, and to include a wider range of experimental settings, for example, hydroponic cultivation and petri dish experiments. As we found that an important difference between settings is the acclimation strategy to face the effects of the stress, we do not believe it would be suitable to combine several species within the same meta‐analysis, as innate variations in acclimation mechanisms (Cheeseman 2014) would hinder the detection of differences in acclimation patterns between settings.

Because of the challenges associated with phenotyping in the field (Pauli et al. 2016; Hu and Schmidhalter 2023), many of the phenotyping platforms where abiotic stress screening is performed are located in GHs (Tardieu et al. 2017). If we want indoor phenotyping results to translate optimally to agricultural conditions, we need to assess the extent to which the experimental setting skews the effect of stress alleviating treatments and the comparison of tolerance between genotypes, as was observed between root phenotypic platforms (Nguyen et al. 2024) and between field and hydroponics conditions (Tavakkoli, Fatehi, et al. 2012). This would avoid wasting time and money on facilities and experiments that cannot identify the genetic or management interventions we need for food security under climate change. An important part of this work will be to identify which aspects of the controlled environment are responsible for differences in abiotic stress response, which may require more experiments that, like ours, directly compare experimental conditions in a systematic way.

### 4.3 Considering Experimental Settings to Improve the Translatability of Abiotic Stress Experiments

Based on 456 articles annotated during our meta‐analysis screening, we found wheat salt stress experiments were carried out 38% of the time in a GH, 21% in the field, 12.5% in OPs, and just 7% in CCs, while in 22% of articles, the experimental setting was either missing from the methods or unclear. The poor representation of CC experiments is probably due to the fact that most of the salt stress studies in that setting use hydroponic systems, which were excluded from our meta‐analysis, as already known to be a poor representation of salt stress in the field (Tavakkoli, Rengasamy, et al. 2010; Tavakkoli, Fatehi, et al. 2012). The high proportion of studies omitting experimental setting from the description of experiments is evidence of how much the importance of this methodological factor is underestimated. Of those studies that described experimental settings, nearly half of wheat salt stress experiments are carried out in GHs, a setting where we found the effect of salt stress to be notably different in extent and pattern from field conditions, which sheds doubts on the relevance of a significant part of wheat salt stress research to agricultural conditions.

Adjusting GH or CC environments to make them more realistic would incur significant costs. As a cheaper alternative, outdoor pot experiments should be considered more often, given that our meta‐analysis shows that for many measurements, these showed similar salt stress responses to field‐grown plants. For our experimental work, we built the necessary rain‐out shelter on a limited budget; for drier countries, well‐designed wire houses, inspired by the ones used for outdoor pot experiments in many studies from Pakistan, would allow many of the benefits of GHs, with protection from the rain with a clear roof (not just so that water input to the pots can be controlled, but also to protect sensors and electrical phenotyping equipment), some exclusion of herbivores, and sufficient airflow to avoid any significant GH effect. Finally, pot experiments should aim for realistic plant density and suitable root space to better represent field conditions (Poorter, Bühler, et al. 2012; Postma et al. 2021).

Now that genotyping can be done cost effectively at high scale and resolution, it is generally considered that the improvement of crop climate resilience requires a combination of advances in phenotyping technology (Tardieu et al. 2017; Mansoor and Chung 2024), applications of artificial intelligence (Farooq et al. 2024), and “omics” approaches (Raza et al. 2024). The latter is preferentially applied under tightly controlled conditions for the sake of replicability, but this urge for replicable experiments is considered by some as counterproductive to translation (Plessis 2023). The enthusiasm about the potential of modern technology may have led to neglecting the fundamental importance of experimental conditions on the reliability of mechanistic studies and screens. Here, we show a clear effect of experimental setting on the pattern of salt stress response in wheat, which is likely to hold true for other abiotic stresses and species, that we hope will bring much needed attention back from technological to methodological progress.

## Conflicts of Interest

The author declares no conflicts of interest.

## Peer Review

The peer review history for this article is available in the Supporting Information for this article.

## Supporting information


**Data S1** Peer Review.

**Figure S1** Photographs of the experimental work.

**Table S1** List of the publications used in the meta‐analysis.

**Table S2** Monthly average, maximum and minimum temperature (°C) for greenhouse (A) and outdoors (pots and raised beds; B) conditions.

**Table S3** Composition of Instant Ocean as percentage of weight.

## Data Availability

The experimental data are available at https://doi.org/10.5281/zenodo.14210102.
