# Supplementary material for: Is My Stress Out of Place? Bread Wheat Response to Saline Stress Varies in Pattern and Extent Across Experimental Settings
Source: Plant Direct. 2025 Jul 2;9(7):e70088. doi: 10.1002/pld3.70088 (PMC12222186; doi:10.1002/pld3.70088)
Supplement: Supplementary file 2 — Figure S1 Photographs of the experimental work. [file PLD3-9-e70088-s004.pdf]

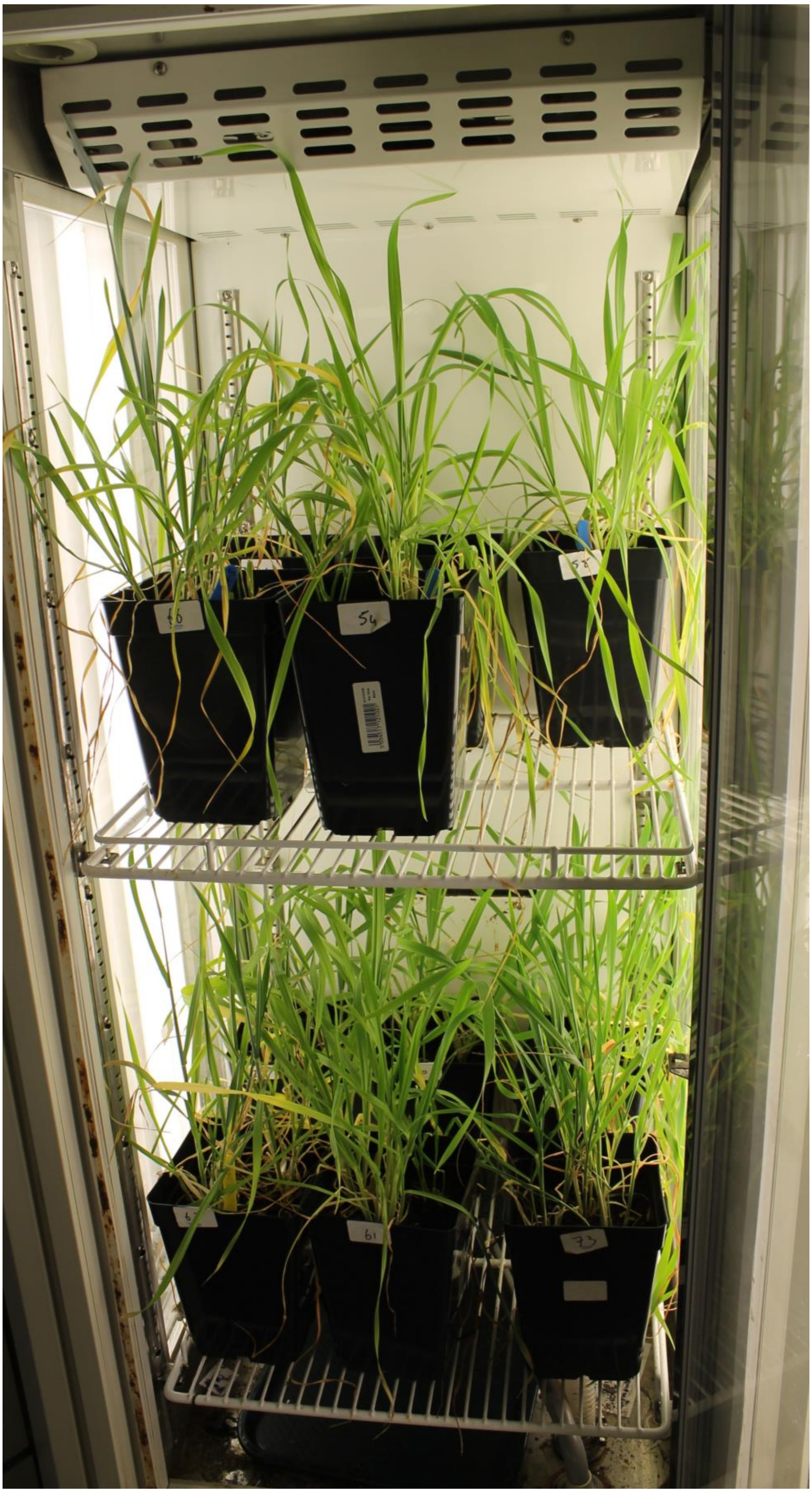

Supplemental Figure S1 A
Climate chamber experiment

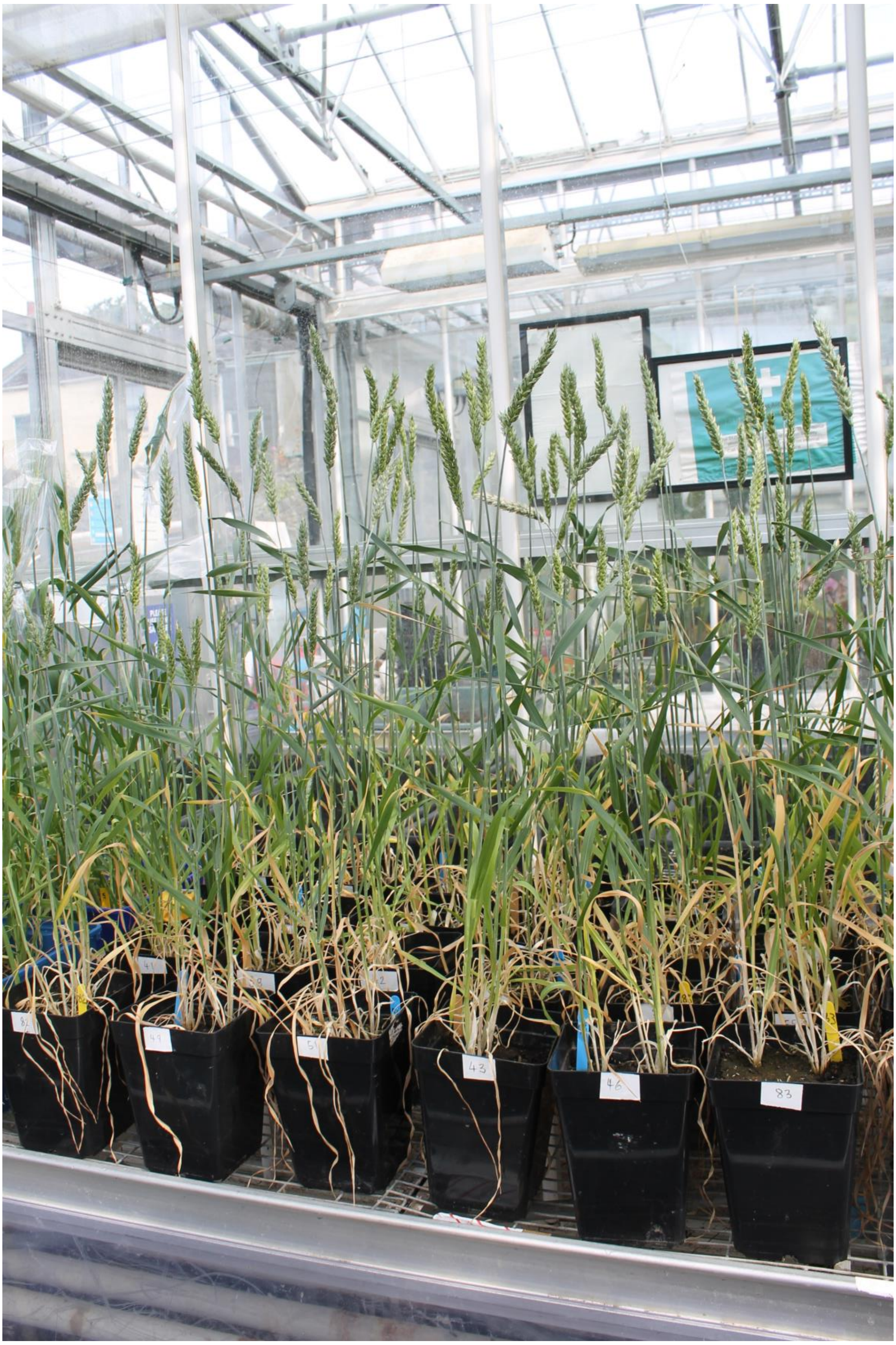

Supplemental Figure S1 B
Greenhouse experiment

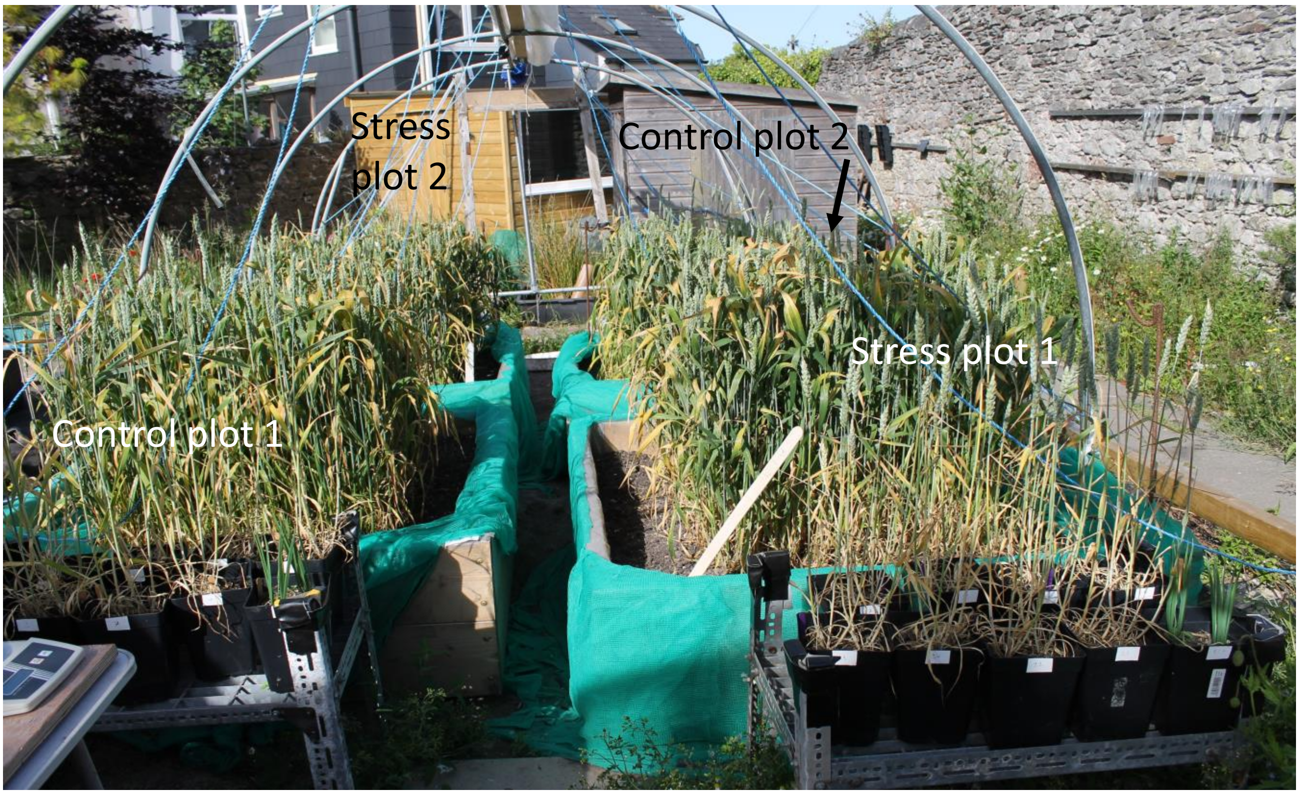

Supplemental Figure S1 C
Raised beds and outdoors pots experiments

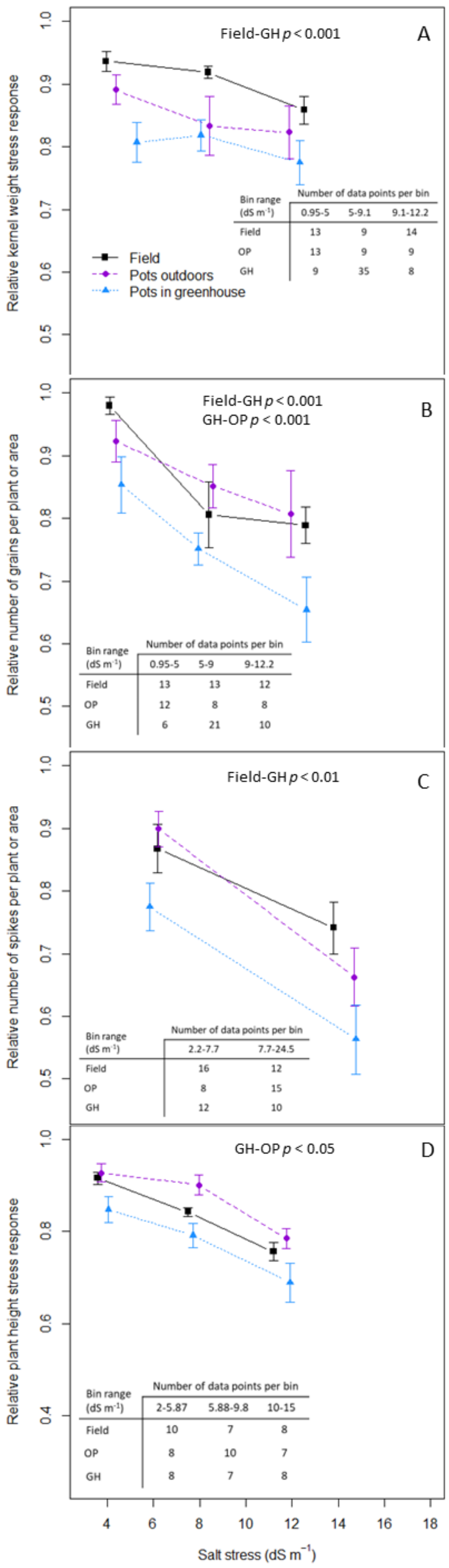

Supplemental Figure S2. Meta-analysis of salt stress response of yield components and height three experimental settings and across different levels of salinity. Ratio of kernel weight in saline stress conditions to control conditions (A). Ratio of number of grains (per plant or per m^2) in saline stress conditions to control conditions (B). Ratio of number of spikes (per plant or per hectare) in saline stress conditions to control conditions (C). Ratio of height in saline stress conditions to control conditions (D). Field: black squares; pots outdoors: purple circles; pots in a greenhouse: blue triangles. Number of data points in each bin of salinity presented in the tables within each plot; statistical analysis for pair-wise comparison of settings included at the top of each plot, obtained with Mann-Whitney *U* test; error bars represent the standard error.

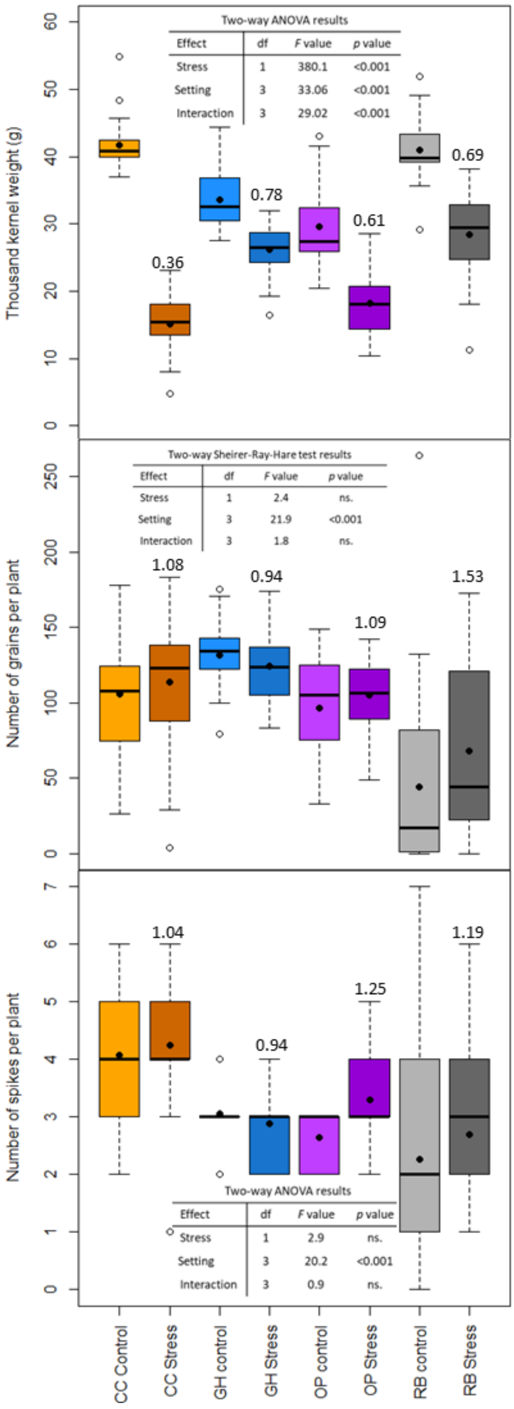

Supplemental Figure S3. Yield components for wheat subjected to salt stress in four experimental settings. Thousand kernel weight (A). Number of grains per plant (B). Number of spikes per plant (C). Number of replicates per setting-treatment group over 17 for CC, GH and OP, over 29 for RB; median represented by black line and mean by dark dot, the box represents the first and third quartile of the data and the whiskers the range of the data, excluding outliers; the number on top of stress boxes is the relative value of the variable (ratio of stress to control values). Statistical analysis included in each plot. CC = climate chamber, orange, GH = greenhouse, blue, OP = outdoors pots, purple and RB = outdoors raised beds, grey; control: light shade; saline stress: dark shade.
