# Supplementary material for: Is My Stress Out of Place? Bread Wheat Response to Saline Stress Varies in Pattern and Extent Across Experimental Settings
Source: Plant Direct. 2025 Jul 2;9(7):e70088. doi: 10.1002/pld3.70088 (PMC12222186; doi:10.1002/pld3.70088)
Supplement: Supplementary file 4 — Table S2 Monthly average, maximum and minimum temperature (°C) for greenhouse (A) and outdoors (pots and raised beds; B) conditions. [file PLD3-9-e70088-s003.pdf]

Supplemental table S2. Monthly average, maximum and minimum temperature (°C) for greenhouse (A) and outdoors (pots and raised beds; B) conditions.

A

Greenhouse

Month	Mean	Mean daily min	Mean daily max	Monthly min	Monthly max
February (from 17th)	11.4	7.85	16.2	7.7	17
March	11.2	6.7	17	4.8	24.4
April	12.3	7.5	17.5	4.6	24.6
May	14.8	9.1	19.4	7.8	35.8
June (until 27th)	17.8	11.2	26.5	7.7	33.3

B

Outdoors

Month	Mean	Mean daily min	Mean daily max	Monthly min	Monthly max
February (from 17th)	8.6	5	10.8	2	12
March	9.8	6.5	13.5	2.1	18.9
April	11	7.2	15.5	-0.2	22
May	14.1	10.2	18.6	5.9	22.6
June	16.4	11.8	21.1	6.6	27.4
July	19.3	14.4	24.4	9.7	35.6
