# Supplementary material for: Is My Stress Out of Place? Bread Wheat Response to Saline Stress Varies in Pattern and Extent Across Experimental Settings
Source: Plant Direct. 2025 Jul 2;9(7):e70088. doi: 10.1002/pld3.70088 (PMC12222186; doi:10.1002/pld3.70088)
Supplement: Supplementary file 5 — Table S3 Composition of Instant Ocean as percentage of weight. [file PLD3-9-e70088-s001.pdf]

Supplemental table S3. Composition of Instant Ocean as percentage of weight.

ion	% weight
Cl^-	47.4700
Na^+	26.2800
SO_4^{2-}	6.6020
Mg^{2+}	3.2300
Ca^{2+}	1.0130
K^+	1.0150
HCO_3^-	0.4910
B	0.0150
Sr^{2+}	0.0010
H_2O	13.8800
