# Supplementary material for: Is My Stress Out of Place? Bread Wheat Response to Saline Stress Varies in Pattern and Extent Across Experimental Settings
Source: Plant Direct. 2025 Jul 2;9(7):e70088. doi: 10.1002/pld3.70088 (PMC12222186; doi:10.1002/pld3.70088)
Supplement: Supplementary file 1 — Data S1 Peer Review. [file PLD3-9-e70088-s002.pdf]

Plant Direct
Open Access

Is my stress out of place? Bread wheat response to saline stress varies in pattern and extent across experimental settings

Dr. Anne Plessis

Decision letter round 1

March 2, 2025

Dr. Anne Plessis

Plymouth University

Plymouth

United Kingdom

RE: Is my stress out of place? Experimental setting affects bread wheat response to saline stress

Dear Dr. Plessis:

Thank you for submitting to Plant Direct. All required reviews have been returned and we have now finished our evaluation of your manuscript. In light of the reviewers' and editor's comments, further revisions are needed before the paper can be accepted for publication in Plant Direct.

Please view the editors' and reviewers' comments below and use their suggestions as a guide while you work on your revision. Overall, I think they both found the length and complexity to be distracting, so you may considering focusing the work to make your main point. While they suggest new experiments, these are not necessarily requires if you can make express any caveats or limitations, and suggest future work.

When uploading the revised version of this article, please be sure to include the following:

-A word document that contains your response to the reviewers. You should respond to each reviewer comment and note the changes made to the manuscript. If you do not agree with a reviewer's comment and choose not to make a suggested revision, please explain why. Please try to

provide as complete an answer as possible to each reviewer's criticisms in the "response to reviewers" section.

-A tracked changes document

- A clean version of the latest version of the manuscript

To upload your revision, please click the link below.

https://plantdirect.msubmit.net/cgi-bin/main.plex?el=A1Lr2mD3A5iTJ6I3A9ftdQOH5TPfCnXvFLbooU7cXkgZ

In order to provide as timely a service as possible, we ask that your revision is resubmitted within three months after receipt of this request. If an extension is needed, please send a request, along with a brief explanation, to the editorial office at plantdirect@wiley.com .

Thank you very much for giving us an opportunity to review your work. I look forward to receiving the next version.

Sincerely,

Larry York

EIC, Plant Direct

-- Reviewer comments:

Reviewer #1:

Plant Direct
Open Access

The Authors present a meta-analysis and a self-conducted study on the effects of salt stress on various plant traits. The focus is, however, not on the effect of salt stress, but the effect of the environment (greenhouse, outdoor, field).

The article is a very long read, with a weak conclusion. I found it way to much work to understand it and follow it. It would greatly benefit from a more precise, information dense writing style. At the end, the reader does not get much insights into salt stress effects (which I had hoped for) as the focus is strongly on the environmental effect. But this environmental effect is not solidly compared to what others reported and seems to drown in speculations about interactions between salt stress and temperature and other factors. These speculations are however not analyzed in the meta-analysis as all the data is analyzed by 'setting' and salt stress intensity, rather than reported temperature, light etc. The overall conclusion is that it would be better to do experiments outside.

Although setting has an overall effect on plants, this was already known from other meta-analysis. The question is if it would change the conclusions of a study (e.g. is the interaction between salt stress and setting strong), and this is not convincingly shown. It seems that indoor experiments might exacerbate the relative stress effect somewhat (not necessarily the absolute effect). From this, you might even conclude the other way around, as inside seems to give you a better stress response and presumably better repeatable results with stronger stats.

- The stats in generally fall short see several comments below.

- The methodology is vague in several places. I could not repeat this.

- The figures can not really be understood without reading a lot and looking up tables and flipping back and forth to compare results. It is basically impossible to compare the meta-analysis quantitatively to the presented experiment.

- It is not clear to me what the presented experiment adds to the meta-analysis, other than the time series, which support the idea of duration of the experiment being important. But maybe this is a false notion as during the experiment the salt concentration was slowly increased.

Title:

Too vague/general to my liking. Experimental settings always matter for all experiments. The main goal of meta-analysis is to see the big picture where the details matter less?

Highlight:

OK, but what are 'real' agricultural conditions. I presume they are extremely variable. And similar in what respect?

Abstract:

L30 It is not so clear from the abstract how greenhouse and field conditions can be compared at all, on the basis of similar salt concentrations, similar exposure time etc?

L33 I am not so sure I would follow your recommendation. Are strong effects not wanted? The question is if one would draw the wrong conclusion. That is, if a gene that confers salt tolerance found in the greenhouse, has no effect in the field. Similar for treatment combinations. The absolute effect here is rather unimportant, what matters is the relative effect.

Introduction

L 46-105 This could be condensed to a couple of sentences.

L 66 Maybe so, but my feeling is that you are purposely understating progress in salt-tolerance research.

L 105 It is very easy to find discrepancies among experiments, but I am sure there also plenty that claim that the work was repeatable. Last, how often were results from field experiments repeatable? How well does the yield of cultivars on a salt stress field correlate to that of the same cultivars grown in a different year or salt stress field?

L 118 Plant density, soil substrate, soil volume, soil temperature vs shoot temperature. I can think of plenty more. But they would also vary from field to field and year to year.

L124, Yes, but more likely to have the same when comparing controlled vs controlled than when comparing field vs field, or isn't that the point of having controlled experiments?

L124 The step controlled=artificial and field=natural is a bit too easy, but 'field' is not the opposite of 'artificial'.

Methods

L 141-143. Excluding....where....were included. I am confused. The measurements were an exclusion criteria? Motivate the choices.

L 155 Criteria which must be made clear and explicit.

L 159 Only cultivars? Or other genotypes as well?

L 161 That seems a rather arbitrary criteria.

L 164 Sodium stress? What about pH? Many salt fields are alkaline.

L 204 ...assuming NaCl was the dominant factor in EC. How was then the EC computed for the low NaCl controls? Also, explicitly give the calculation, or a reference for it.

L 211 Also give insight into the number of studies disqualified because of previously described rules. It matters if this is 50% or only 5% of the total. Insight into what rules removed the most studies might be useful.

L 215 What was done if the NaCl was combined with other treatments or genotypes? Were these averaged, or taken as separate points?

L 217 Other representations may be useful - for example was there a 'dominant' author or lab? The R bibliometric package can be helpful to make these things insightful.

L 224 Stress increased with stress? Choose better wording.

L 227 length → duration?

L 230 You basically compute an integral over time. So 'stress integral', 'stress sum' , or 'stress duration' as in 'light integral', 'temperature sum', or 'leaf area duration' might be clearer.

L 230 Important to show that this does not great a bias towards field experiments being longer and thus having a greater stress gauge.

L 231 'minus' is not a relative measure. What was relative to what? Consider providing a simple equation.

L 239 Binning sounds arbitrary. There are formal procedures for this, better make this more clear, or use a true grouping algorithm.

L 239,244,figures The binning looks clean on the figures, but still the linear lines are disturbing. I would much prefer to see a point cloud with a fitted line as suggested by Poorter et al. On how to construct dose-response curves.

L 239 the binning led to heavily unbalanced designs (see table 4 particularly). Given interaction effects, this can not be done with a normal anova in R as it only supports mildly unbalanced designs. It seems you need mixed linear models, possibly with study as a random factor, although maybe the scaling took care of that. Please comment in the stats section, and or change stats.

L 251 At this stage it is not clear why you add an experiment. The motivation for experimentation over meta-analysis should be spelled out in the introduction.

L 261 The wording suggest it wasn't closed for light precipitation. What was the criteria of 'heavy'?

L 269 3 kg in a 4.5 liter pot seems to give a very low bulk density.

L 290 how do you integrate this changing salt level over time to compute the 'stress gauge'?

L 357 'not normal' based on what criteria? Ratios of two normal distributed measures are Cauchy distributed, see ratio distribution. The step from not normal to non-parametric is rather large and actually not always necessary. For example R can do GLM perfectly with a cauchy distribution.

Results

Fig 1 Since these are bins, shouldn't the points also have horizontal error bars? Fig legend is not clear on the presented error bars are these standard error of the means? Figure does not indicate significant differences between lines or points. Panel C seems redundant after A and B as A=B*C.

Table 1 Unusual and confusing presentation of Anova table results lacking DF. Is stress level categorical? Why is there no post-hoc result for stress level. Or is it to be a linear response (linear regression). Left and right side of the table have nothing to do with each other, this should really be split into two tables. It took me way to much time to understand this. Ideally, the info is integrated into a better fig 1.

Other fig and tables: See remarks table 1 fig 1. In addition, make the axes start a 0, and ideally have the same scale so meta analysis and experimental study are easier compared. Consider marking in the meta-analysis the dots of the experiment.

L 367: Rather complicated wording 'a higher negative response is visible from a line that is running lower in the graph'.

L 435: This interpretation of the results assumes that the relative stress levels presented are significantly different from 1 (the reference), but the stats actually do not show this.

L 456 not the sodium in pots but the sodium concentration in the shoot of plants grown in a greenhouse vs climate chamber.

Table 4 the binning here seems to become extra problematic as the groups are few and wide and very unbalanced.

L 503 since this is a single study, could you build confidence first that the treatments were comparable with respect to salt levels?

L 503 I think describing the environmental conditions first would really help. Besides the salt levels, I am particularly interested in amount of water, light levels (DLI), nutrient levels etc. It would be interesting to know how these differences in environment in this particular experiment relate to those in the meta-analysis. Maybe you are a much 'better' experimenter than the average research in the meta-analysis.

L 503 From a readers perspective, it might be more interesting to provide detailed insights from a particular study and then expand using meta-analysis, than the other way round. After all, why is one experiment interesting after a 100 have been analyzed?

Fig6, where are the stats? Reading them from a fig legend is very hard. Besides, a two-way Scheirer-Ray-Hare test does not provide the level of differences compared to a normal post-hoc with letters. Last, time series are not independent, and need appropriate treatment of repeated measurements or growth curve fitting.

L 508 Raised beds are definitely not field. Do not confuse the two.

Discussion

L 595 did your study include all the 16 studies of Cheng et al? Provide the number of overlapping studies.

L 597 A bit too much of clapping on your own shoulder, I would say. It seems to insinuate that we can just forget about Cheng. Maybe your approach can be more positive.

L 605 I agree that crop models generally include sink limitations poorly. This is not only true for the seed number, but also for other organ counts.

L 607 Substantial, complex, they are such vague words. They really put my off reading.

L 608 I keep stumbling over 'setting'. Why not use 'environment'?

L 617 'specificity' what am I to do with that. Does everything matter?

L 618 You just bashed Cheng on having only 16 studies, and now you are happy with 10. It feels a bit unfair.

L 623 - 624 Now it becomes interesting. But why not show the temperature difference among the studies? Is this pot or air temperature, and I presume outside the fluctuation is much higher. Reading this I looked up Poorter's Pampered inside pestered outside paper. It has temperature differences of at most 5 degrees Celsius between inside and outside. Is that really going to matter?

L 633 Root mass fraction or root/shoot ratios?

L 638 Before going on, it would have been helpful if you discussed environmental factors other than temperature. Light surely explains also some of the results?

L 647 Only means that your treatment was relatively mild? Again, it would be good to know where on the gauge and salt scale your experiment sits compared to the others analyzed in the meta-analysis.

L 655 Good, how does salt-stress affect time to flowering?

L 633 Work out plant density more, like you did with the other environmental factors.

L 666 I find this section uninteresting and I suggest removing or shortening it drastically. We can learn nothing from how a particular experiment deviates from the average. It is all speculation. The particular experiment should ideally agree with the meta-analysis and be used to fill in blanks where the meta-analysis falls short.

L 702 This section states a lot of what was already said by others with respect to lab to field translation. It tends to ignore the motivation to do things in the lab under controlled conditions and thereby fails to convince. A major argument for having controlled conditions is repeatability. The results presented show that indeed the salt stress response might quantitatively depend on 'setting' but qualitative, the response is amazingly similar.

Conclusion

The text is more a summary with references and argumentation, than a listing of the major findings of the article. Suggestions for further research belong in the discussion.

Reviewer #2:

Plant Direct
Open Access

See pdf (please find PDF attached and let office know if no PDF is included by replying to this email and yorklm@ornl.gov to include EIC)

Decision letter round 2

June 19, 2025

Dr. Anne Plessis

Plymouth University

Plymouth

United Kingdom

MSID: 2024-01685-TWR1

MS TITLE: Is my stress out of place? Bread wheat response to saline stress varies in pattern and extent across experimental settings

Dear Anne:

I am pleased to inform you that your manuscript "Is my stress out of place? Bread wheat response to saline stress varies in pattern and extent across experimental settings" has been accepted for publication in Plant Direct. I appreciate the huge amount of work that has gone into the extensive revision, as well as the careful and thorough commentary on the changes.

Your article will appear online in the next available issue of Plant Direct. To ensure your article gets published as quickly as possible, please pay attention to the steps detailed below. We have found that most of the delays happen at this stage, especially at the payment stage, so please respond as quickly as possible when prompted.

License Agreement: Once your article has been accepted it will move to Production and undergo admin and file checking - you may receive an email with any queries we have at this stage. When all required items are received by the publisher and queries resolved, the corresponding author will receive an email from Wiley's Author Services system which will ask them to log on at https://authorservices.wiley.com/bauthor and will present them with the appropriate license for

completion. Your article cannot be published until both the signed license agreement and payment of the article fee have been received.

Payment of the Open Access Article Publication Fee: All articles published in Plant Direct are fully open access: immediately and freely available to read, download and share. Plant Direct charges a publication fee to cover the publication costs. The corresponding author for this manuscript should have already received a quote from the payments team (cs-openaccess@wiley.com) with the estimated article publication fee; please email cs-openaccess@wiley.com if this has not been received. The corresponding author should log on to the Wiley Author Services site, where the publication fee can be paid by credit card or an invoice. Pro Forma can also be requested. Payment of the publication charge must be received before the article will be published online.

Proofs: You will have the opportunity to look over your paper once more when you receive the author proofs for your article. The proofs will be with you in approximately two weeks. Please note that, in addition to publishing reviewer comments, the author's responses to review comments will also be published alongside the final version of the paper. If you would not like the author's responses to be published, please contact the editorial office at plantdirect@wiley.com .

Promotion of your article: You can help your research get the attention it deserves! Check out Wiley's free Promotion Guide for best-practice recommendations for promoting your work at www.wileyauthors.com/eeo/guide. And learn more about Wiley Editing Services which offers professional video, design, and writing services to create shareable video abstracts, infographics, conference posters, lay summaries, and research news stories for your research at www.wileyauthors.com/eeo/promotion.

Thank you again for your contribution to Plant Direct. If you have any questions, feel free to contact the editorial office at plantdirect@wiley.com .

Best wishes,

Jonathan

Jonathan Ingram

Editor, Plant Direct

Response to reviewer round 1

Editor 1. consider focusing the work to make your main point Thanks to the comments of the reviewers, I have now moved the emphasis in the interpretation of the results on the difference in pattern rather than extent of the salt stress response, which I believe provides a more impactful message. While doing this, I have reduced the number of figures from 7 to 5 and removed all of the tables, leading to the text of the results going from 1179 to 952 words. The introduction is also a bit shorter now (from 1207 to 1145 words). Despite adding limitations and future work to the discussion, it is quite similar in length to the original version (from 2425 to 2415 words). 2. express any caveats or limitations, and suggest future work The new version of the discussion now contains a section entitled "Challenges and future work in understanding the consequences of experimental settings on abiotic stress studies" that covers limitations and some more future work than in the original manuscript. Reviewer 1 General comments: -weak conclusion The new version of the discussion does not include a conclusion anymore. - more precise, information dense writing style I have reduced the number of figures from 7 to 5 and removed all of the tables, leading to the text of the results going from 1179 to 952 words. The introduction is also a bit shorter now (from 1207 to 1145 words). Despite adding limitations and future work to the discussion, it is quite similar in length to the original version (from 2425 to 2415 words). - the reader does not get much insights into salt stress effects This is not the point of this study and I have made this clearer by deleting the part of the discussion that dealt with general findings about salt stress. The research question is about the impact of experimental settings and salt stress here is just used as a case study. - It seems that indoor experiments might exacerbate the relative stress effect somewhat (not necessarily the absolute effect). From this, you might even conclude the other way around, as inside seems to give you a better stress response and presumably better repeatable results with stronger stats. This comment is not that relevant anymore given the new focus on pattern of response and the related interpretation of different mechanisms being used by plants to face salt stress across settings. See also replies below on the replicability aspect. - It is basically impossible to compare the meta-analysis quantitatively to the presented experiment. I have now presented meta-analysis and experimental results in the same section to facilitate this comparison. There was already in the original version of the manuscript a way to compare those results together: the response ratio values provided on the experimental figures as numbers on top of the box plots are directly comparable to the relative stress response values plotted for the meta-analysis. - It is not clear to me what the presented experiment adds to the meta-analysis, other than the time series A section has now been added to the discussion explaining how the two approaches are complementary, each with their own limitations that the other approach makes up for. Title: Too vague/general to my liking. Experimental settings always matter for all experiments. The main goal of meta-analysis is to see the big picture where the details matter less? While the fact that experimental setting matters might be obvious to some researchers, it is not for all, as demonstrated by our data: over 20% of publications not stating the experimental setting of their study. In addition, being aware of the effect of experimental setting is not the same thing as having access to data to evidence this effect. This is well supported by the fact that the meta-analysis by Poorter et al (2016), about the general effect of experimental setting has been cited 369 times according to Web of Science. I agree that some more precision in the title is beneficial, and I have therefore rephrased the title in the new version of this manuscript. Highlight: OK, but what are 'real' agricultural conditions. I presume they are extremely variable. And similar in what respect? An highlight is not required by Plant Direct, I have therefore removed it. Abstract: L30 It is not so clear from the abstract how greenhouse and field conditions can be compared at all, on

the basis of similar salt concentrations, similar exposure time etc? I have added precision on this point in the abstract: “comparing responses relative to control conditions over similar ranges of salt stress intensity”. L33 I am not so sure I would follow your recommendation. Are strong effects not wanted? The question is if one would draw the wrong conclusion. That is, if a gene that confers salt tolerance found in the greenhouse, has no effect in the field. Similar for treatment combinations. The absolute effect here is rather unimportant, what matters is the relative effect. I have fully taken this comment into account, and the new version of the manuscript now focuses on the difference in acclimation strategy. I have modified the abstract accordingly, so this comment does not apply anymore. Introduction L 46-105 This could be condensed to a couple of sentences. I have cut down the first paragraph on the food security context as it may not be essential, but I believe that a strong argument for the wide-ranging translation problem is crucial to convince the reader of the value of this study. In addition, as this is a method paper, the research context is very relevant to understand the impact of current experimental methodology, which is why I have kept the second paragraph as it is. I also want to point out that the section highlighted here as needing to be “condensed into a couple of sentences” makes up 851 words out of a 1207 words introduction (i.e. 70% of the introduction). Is the suggestion here to decrease the introduction by more than half? L 66 Maybe so, but my feeling is that you are purposely understating progress in salttolerance research. There is no question that there is constant progress in identifying genes involved in salt tolerance, my point is about how this translates into improved varieties. The sentence being questioned here is only about breeding and is not making a particularly controversial point, just stating that two main cereals have been more difficult to breed for abiotic stress tolerance than other cereals. In addition, the reviews cited to support this point are not cherry picked, I believe they reflect the opinion of the researchers involved in this translational work, rather than that of the groups that do more fundamental research. L 105 It is very easy to find discrepancies among experiments, but I am sure there also plenty that claim that the work was repeatable. Last, how often were results from field experiments repeatable? How well does the yield of cultivars on a salt stress field correlate to that of the same cultivars grown in a different year or salt stress field? I agree that it is likely that some work is repeatable between settings, and there is nowhere in my manuscript where I say that it is not possible to replicate results between controlled and field conditions. However, the justification for my study is that it is not always the case and, at the moment, it is unclear to what extent this affects abiotic stress experiments, but this is important to know if we want to improve translation to more resilient crops. To answer the second point of this comment, here, the emphasis is on understanding mechanisms of stress resilience and identify ways to improve crops, not about replicating experiments. Understanding year to year differences in the field actually involves studying the impact of the factors like temperature and precipitation, that would vary between years, and acquiring that knowledge is likely to be more fruitful using studies done in realistic conditions than in very artificial conditions. Therefore, I don’t think the fact that each field study differs from the next invalidates the rationale of this study. L 118 Plant density, soil substrate, soil volume, soil temperature vs shoot temperature. I can think of plenty more. But they would also vary from field to field and year to year. Soil volume and root temperature were already mentioned in a previous sentence centred on the effect of pot cultivation. Airflow intensity and quality (outdoors wind and greenhouse ventilation have different patterns) sets experimental settings apart in a distinctive way, while soil substrate is not characteristic of one particular setting, soil can be used in any setting, so I don’t think it needs to be mentioned here. Plant density, on the other hand, is more integral to the growth conditions of

specific environmental settings and accordingly, a sentence has been added in the introduction to include this factor with those varying between settings. L124, Yes, but more likely to have the same when comparing controlled vs controlled than when comparing field vs field, or isn't that the point of having controlled experiments? As pointed out in a previous publication, replicability under controlled conditions is not necessarily a valuable goal when the purpose of the research is translation to the real world (Plessis, 2023). That said, I agree that this sentence is not making a very meaningful point, so I have modified it to better reflect the focus of this new version on the mechanisms of stress tolerance. L124 The step controlled=artificial and field=natural is a bit too easy, but 'field' is not the opposite of 'artificial'. The word "artificial" has been removed from this sentence. Methods L 141-143. Excluding....where....were included. I am confused. The measurements were an exclusion criteria? Motivate the choices. To keep the results of this study to a reasonable volume and level of complexity, it was necessary to limit the number of settings and measurements included. I decided to focus on the most novel aspects of experimental conditions, which is why hydroponics was excluded, as its impact has already been studied. In vitro studies are generally very short and not comparable enough. The choice of measurements to include was motivated by the type of measurements most often carried in field environments, as the most informative comparison is between field and other settings to understand the contribution of experimental setting to the lack of translation of abiotic stress studies to the improvement of yield resilience to abiotic stress. A sentence has been added to make this clearer L 155 Criteria which must be made clear and explicit. This has been done now at the beginning of the meta-analysis section of the methods, with each of the five criteria listed and more clearly defined. L 159 Only cultivars? Or other genotypes as well? No other information beyond species and cultivar was provided in the studies collected that could have helped identify that the study organism was indeed bread wheat. L 161 That seems a rather arbitrary criteria. Unfortunately, the studies collected included a wide range of distances (as far as 600 km) between control and saline fields, so a criterion had to be chosen and this will inevitably introduce a level of arbitrary choice. It was estimated that 50 km was close enough that the soil and climate would be similar enough for comparison. Similarity in climate and soil was actually stated in some studies with distances slightly below 50 km. L 164 Sodium stress? What about pH? Many salt fields are alkaline. Alkaline-saline stress was exclude, which has now been made clear in the criteria. L 204 ...assuming NaCl was the dominant factor in EC. How was then the EC computed for the low NaCl controls? Also, explicitly give the calculation, or a reference for it. Yes, I did check that NaCl was the dominant factor in the case of natural soils where ion composition of the soil was provided. In the vast majority of studies where NaCl molar concentrations are provided as the level of stress, the NaCl solution is applied in addition to any existing ions (e.g. nutrient solution or pre-existing salinity in the soil). Given that I calculated stress intensity by subtracting the EC of control conditions to the EC of the stress conditions, any other source of conductivity can be ignored as similar in control and stress conditions and therefore "cancelled out" during the calculations of stress level. Calculation of ECs are now described more precisely. L 211 Also give insight into the number of studies disqualified because of previously described rules. It matters if this is 50% or only 5% of the total. Insight into what rules removed the most studies might be useful. These numbers have been added when possible. L 215 What was done if the NaCl was combined with other treatments or genotypes? Were these averaged, or taken as separate points? This is explained earlier in this section of the methods. L 217 Other representations may be useful - for example was there a 'dominant' author or lab? The R bibliometric package can be helpful to make these things insightful. I

did not find any R package with this name. I did find a package called “bibliometrix”, however, it is designed to analyse bibliometric data directly exported from a database (https://www.bibliometrix.org/vignettes/Data-Importing-and-Converting.html), not list of studies resulting from a combination of Rayyan and manual screening like mine. I found no way to import the data for my set of studies in the right format that could be analysed by this package. L 224 Stress increased with stress? Choose better wording. The sentence referred to here actually states that the EFFECT of the stress (not the stress itself) increased with the intensity of the stress. L 227 length → duration? Yes. This has been corrected. L 230 You basically compute an integral over time. So 'stress integral', 'stress sum' , or 'stress duration' as in 'light integral', 'temperature sum', or 'leaf area duration' might be clearer. This is a valid point, it does make it clearer to call this calculation “stress integral” therefore this is have been modified in the text and figures. L 230 Important to show that this does not great a bias towards field experiments being longer and thus having a greater stress gauge. As displayed on figure 3, the stress gauge was only used to compare climate chamber and greenhouse experiments, and only relatively short-term experiments as they are all limited to the vegetative stage as stated L237. L 231 'minus' is not a relative measure. What was relative to what? Consider providing a simple equation. This has been done. L 239 Binning sounds arbitrary. There are formal procedures for this, better make this more clear, or use a true grouping algorithm. Binning was not done in an arbitrary way at all, but did involve comparing different options to identify a set of bins that would make settings as comparable as possible (i.e. average stress intensity within a given bin as close as possible across settings) while avoiding having bins with fewer than 5 data points. In some cases, it was done using a simple algorithm that started by splitting the data from the setting with smallest number of data points equally into the highest number of bins possible, reducing the number of bins if the results did not group stress intensity averages similar enough. If this still did not work, bins limits were set manually by visually inspecting the distribution of the stress intensity over the different settings, optimising the split according to the criteria outlined above. As can be observed in the new versions of the figures, this process led to reasonably close stress intensity averages for each bin across settings. I did not find any grouping algorithm that would create suitable bins for my criteria automatically: most clustering algorithms have very different purpose than what I was trying to do here. L 239,244,figures The binning looks clean on the figures, but still the linear lines are disturbing. I would much prefer to see a point cloud with a fitted line as suggested by Poorter et al. On how to construct dose-response curves. The method outlined by Poorter is not designed for comparison between groups. See” General answer on the data analysis approach and statistical methods used” at the end of this document on why binning is more appropriate than a continuous dose-response curve for this particular meta-analysis. L 239 the binning led to heavily unbalanced designs (see table 4 particularly). Given interaction effects, this can not be done with a normal anova in R as it only supports mildly unbalanced designs. It seems you need mixed linear models, possibly with study as a random factor, although maybe the scaling took care of that. Please comment in the stats section, and or change stats. See” General answer on the data analysis approach and statistical methods used” at the end of this document on how the statistical analysis was modified to account for unbalanced design. L 251 At this stage it is not clear why you add an experiment. The motivation for experimentation over meta-analysis should be spelled out in the introduction. Based on the comment of this reviewer, it seems that some clarity is needed on the fact that the meta-analysis does not compile studies like my experiment that compared the same salt stress over different experimental settings, as these are very rare, but a

more unusual approach where the effect of a methodological factor is analysed across experiments (though this has been done in the past, see for example Cheng et al 2021, cited in the previous version of the manuscript, which looked at the effect of species, soil characteristics and irrigation type). This has now been clarified in the introduction. In addition, the benefits of the meta-analysis/experiment combination is justified in the discussion, showing that they are complementary by outlining different advantages and limitations for each approach. I have also noted that, in contrast with this comment, the other reviewer praised the use of two complementary approaches. L 261 The wording suggest it wasn't closed for light precipitation. What was the criteria of 'heavy'? It is difficult to have a clear criterion when decisions are based on weather forecast rather than records of weather conditions, but the general rule was to close the shelter when a period of rain that would amount to more than 2 mm of precipitation had been forecasted. L 269 3 kg in a 4.5 liter pot seems to give a very low bulk density. As stated the pots were 3 L, not 4.5 L: the surface was larger at the top (where dimensions are given) than at the bottom, which is why the volume cannot be calculated from those dimensions. L 290 how do you integrate this changing salt level over time to compute the 'stress gauge'? The stress gauge/integral was only used in the meta-analysis, not in the experiment, where the start of the salt stress was when the lowest concentration of salt was introduced to the plants. L 357 'not normal' based on what criteria? Ratios of two normal distributed measures are Cauchy distributed, see ratio distribution. The step from not normal to non-parametric is rather large and actually not always necessary. For example R can do GLM perfectly with a cauchy distribution. The normality of the residuals of the ANOVA, not of the data itself, is tested using a Shapiro Wilk test (this information has been added in the manuscript). We have noticed little loss of statistical power between the ANOVA and the Scheirer-Ray-Hare test, so this not of concern. Results Fig 1 Since these are bins, shouldn't the points also have horizontal error bars? Fig legend is not clear on the presented error bars are these standard error of the means? Figure does not indicate significant differences between lines or points. Panel C seems redundant after A and B as A=B*C. Including horizontal error bars would be relevant if the analysis included comparing the different bins with each other, but this is not the case anymore. I preferred providing information on the size and content of the bins in the tables included in the figures as I found that horizontal error bars impaired the legibility of the results as the different lines are often close to each other. Information on the error bars has been added to the figure legends. This new version does not include yield components (B and C) within the main figures anymore. Table 1 Unusual and confusing presentation of Anova table results lacking DF. Is stress level categorical? Why is there no post-hoc result for stress level. Or is it to be a linear response (linear regression). Left and right side of the table have nothing to do with each other, this should really be split into two tables. It took me way to much time to understand this. Ideally, the info is integrated into a better fig 1. In the new version, there is no more ANOVA and the results of the statistical tests are presented only for significant difference within the figure so those confusing tables do not exist anymore. The comments on the statistical analysis of the effect of stress intensity do not apply anymore as that analysis has been removed for clarity and I now focus exclusively on the comparison between experimental settings. Other fig and tables: See remarks table 1 fig 1. In addition, make the axes start a 0, and ideally have the same scale so meta analysis and experimental study are easier compared. Consider marking in the meta-analysis the dots of the experiment. Because figures 1 to 4 (of the original version) are presenting relative responses (due to requirements of normalisation between studies, which is the standard of meta-analyses) rather than absolute values of measurements, the meaningful value on the y axis is 1,

which represents the absence of effect of the stress and is as a point of reference on all of the meta-analysis plots. Axes for the experimental data have been changed to start at zero. The meta-analysis and experimental results are presented in different scales, the metaanalysis has to be presented on a relative scale for reasons of normalisation between the studies, while I decided to present the experimental results on an absolute scale because there is no need for normalisation in that case given that the experiments are all comparable to each. Absolute values are more informative as they also allow to show the effect on experimental setting in control conditions. To make comparisons between meta-analysis and experimental results possible, I included response ratios values in the experimental results plots, those are the values can be related to those presented in the meta-analysis plots. I have created a figure for the yield data that includes all the data points of the meta-analysis (see below) and I found this makes the figure more difficult to read and is not informative in the way seeing the datapoints on a box plot can be. L 367: Rather complicated wording 'a higher negative response is visible from a line that is running lower in the graph'. This sentence has been removed from the new version. L 435: This interpretation of the results assumes that the relative stress levels presented are significantly different from 1 (the reference), but the stats actually do not show this. This sentence has been removed from the revised version. L 456 not the sodium in pots but the sodium concentration in the shoot of plants grown in a greenhouse vs climate chamber. This sentence has been rephrased to avoid the confusion highlighted here. Table 4 the binning here seems to become extra problematic as the groups are few and wide and very unbalanced. See" General answer on the data analysis approach and statistical methods used" at the end of this document on how the issue of unbalanced design was addressed in the new statistical analysis. L 503 since this is a single study, could you build confidence first that the treatments were comparable with respect to salt levels? Salt levels were different between pots and the raised beds, as can be expected from the fact that the salt can spread much deeper in the raised beds. The data collected here does not really allow to make the suggested statement, but differences in the distribution of soil salinity between pots and open ground is now mentioned in the discussion. L 503 I think describing the environmental conditions first would really help. Besides the salt levels, I am particularly interested in amount of water, light levels (DLI), nutrient levels etc. It would be interesting to know how these differences in environment in this particular experiment relate to those in the meta-analysis. Maybe you are a much 'better' experimenter than the average research in the meta-analysis. Unfortunately, the data necessary for the calculation of daily light integrals was not collected during these experiments but it would be very similar between RB, OP and GH as these settings were right next to each other. It was not deemed necessary to measure as it is very rarely reported in abiotic stress field studies. All other environmental conditions mentioned in this comment were kept similar between the settings, with the same soil used and same amount of fertiliser provided, soil moisture kept at similar levels. Given that the studies reviewed in the meta-analysis did not attempt to compare the stress across settings, the comment about this experiment being better conducted than the ones in the meta-analysis is difficult to understand. L 503 From a readers perspective, it might be more interesting to provide detailed insights from a particular study and then expand using meta-analysis, than the other way round. After all, why is one experiment interesting after a 100 have been analyzed? Once again, there seems to be a misunderstanding here, as my experiment is of a fundamentally different nature from the ones used in the meta-analysis. See my answer to comment on L 251 from this reviewer for the full justification of why the two approaches are complementary. Fig6, where are the stats? Reading them from a fig legend is very

hard. Besides, a two-way Scheirer-Ray-Hare test does not provide the level of differences compared to a normal posthoc with letters. Last, time series are not independent, and need appropriate treatment of repeated measurements or growth curve fitting. The statistical results are now presented within the figures for the experimental results. Because the research question is whether experimental setting impacts on the effect of salt stress, the result of interest is the presence of a significant interaction between the stress and the setting, which the two-way Scheirer-Ray-Hare test assesses. Knowing differences between groups is not the point here. It would be possible to test for each setting the significance of the effect of the stress, but in that case, what will matter would be whether this varies over the different settings, which is just a more convoluted way of testing for an interaction between stress and setting. As stated in the legend, the statistical test was only carried out for the very last time point, not for the entire time series. L 508 Raised beds are definitely not field. Do not confuse the two. This is right. Reference to field conditions have been removed from the description of the experimental results, replaced with either field-like or raised beds. Discussion L 595 did your study include all the 16 studies of Cheng et al? Provide the number of overlapping studies. This section of the discussion has been removed in the new version of the manuscript. L 597 A bit too much of clapping on your own shoulder, I would say. It seems to insinuate that we can just forget about Cheng. Maybe your approach can be more positive. This section of the discussion has been removed in the new version of the manuscript. L 605 I agree that crop models generally include sink limitations poorly. This is not only true for the seed number, but also for other organ counts. Noted. L 607 Substantial, complex, they are such vague words. They really put my off reading. This subheading has been removed as part of changing the structure of the discussion. L 608 I keep stumbling over 'setting'. Why not use 'environment'? I decided not to use the word environment because this term includes everything the plant is experiencing, not just what is specific to the “facilities” where the plants are being grown, in particular any stress being applied. I considered using “location” instead, but found it to be too easily confused with the geographical location. To make sure it is clear what setting is referring to, I have now made sure to always state “experimental setting”. L 617 'specificity' what am I to do with that. Does everything matter? This sentence has been rephrased to be more precise and is now followed by one of the main “specificity” that could explain the differences. L 618 You just bashed Cheng on having only 16 studies, and now you are happy with 10. It feels a bit unfair. This comparison is not relevant now that the section mentioning Cheng has been removed. I do agree this quite a low number, showing that there are few studies that have looked at differences between settings at the same scale as I have. L 623 - 624 Now it becomes interesting. But why not show the temperature difference among the studies? Is this pot or air temperature, and I presume outside the fluctuation is much higher. Reading this I looked up Poorter's Pampered inside pestered outside paper. It has temperature differences of at most 5 degrees Celsius between inside and outside. Is that really going to matter? Temperature data was not collected as part of the meta-analysis as it is too often missing from field, outdoors and greenhouse studies. Because a majority of studies looking at the effect of temperature tend to use stress conditions much higher (generally around 10 C) than the control conditions, we find 5 C a small difference, but over a long of time, this has big effects on development and physiology. L 633 Root mass fraction or root/shoot ratios? This sentence has been removed from the current version of the discussion. L 638 Before going on, it would have been helpful if you discussed environmental factors other than temperature. Light surely explains also some of the results? Light is unlikely to differ to a great extent between greenhouse and outdoors. The new version of the discussion includes more explanations

for both the difference in extent and in pattern of the response, including planting density, air movement and salt distribution in the soil. L 647 Only means that your treatment was relatively mild? Again, it would be good to know where on the gauge and salt scale your experiment sits compared to the others analyzed in the meta-analysis. This sentence has been removed from the new version of the discussion. The stress intensity in my experiment was actually quite high, as pointed out by reviewer 2. Especially now that the focus has moved on from differences between settings in the extent of the effect to the pattern of the response, it seems superfluous to include a comparison of the extent of the effect of the stress between the two approaches: comparisons of the differences between settings are much more relevant. L 655 Good, how does salt-stress affect time to flowering? I am not entirely sure I understand the point of this question. Unfortunately, flowering time could not be included in the meta-analysis as it is very rarely measured outside of field studies. L 633 Work out plant density more, like you did with the other environmental factors. I am not sure what is meant here, as there is no mention of density at this point in the text. A paragraph on the effect of density has been added, if this was what was suggested. L 666 I find this section uninteresting and I suggest removing or shortening it drastically. We can learn nothing from how a particular experiment deviates from the average. It is all speculation. The particular experiment should ideally agree with the meta-analysis and be used to fill in blanks where the meta-analysis falls short. This comment suggests that the experiment conducted for this study and the ones included in the meta-analysis are the same, while this is not the case. While the studies pooled in the meta-analysis investigate the effect of salt stress in their own specific setting, mine is testing the effect of setting on the effect of salt stress. The meta-analysis and my experiment are the ones asking the same question (the effect of setting) with each a different approach, which is why it is relevant to compare their results; this is different from asking whether my experiment is within the average of the studies making up the meta-analysis. The section has been changed in the new version, hopefully making a clearer point of the complementarity of the two approaches. L 702 This section states a lot of what was already said by others with respect to lab to field translation. It tends to ignore the motivation to do things in the lab under controlled conditions and thereby fails to convince. A major argument for having controlled conditions is repeatability. The results presented show that indeed the salt stress response might quantitatively depend on 'setting' but qualitative, the response is amazingly similar. I do not agree with the last sentence of this statement, as there are clear disparities in the pattern of the salt stress response and I hope that this is better demonstrated in the current version of the manuscript. Regarding repeatability, I have already explained in response to a previous comment that replicability under controlled conditions is not necessarily a valuable goal when the purpose of the research is translation to the real world. A short sentence to that effect has been added to the discussion. Conclusion The text is more a summary with references and argumentation, than a listing of the major findings of the article. Suggestions for further research belong in the discussion. The discussion does not include a conclusion section anymore, as it has been re-written according to the requests of the editor. Reviewer 2 Plessis: Is my stress out of place? This ms is a combination of a meta-analysis and a targeted experiment. The meta-analysis combines a range of separate experiments carried out with bread wheat in growth chambers, glasshouses, pots in the field and in the soil. The experiment focuses on a bread wheat cultivar grown specifically under these four settings. Results show that salinity does not have the same quantitative effect in all 4 conditions. In the meta-analysis, glasshouses conditions stand out as negatively affecting plant growth by salinity. In the experiment, growth chamber and field conditions stimulated shoot weight

at week 7. But at final harvest, salinity affected yield most severely and negatively (-60%) in the growth chamber, whereas in the field salinity actually stimulated yield (+15%). I find this is a difficult ms to review. The author is to be applauded by combining a metaanalysis, from which we only indirectly can derive the effects of Salinity x Growth environment, with an own targeted experiment that focused exactly on that issue. They give rather different results, though, so in the end we still have a somewhat complicated conclusion that setting is important, but with rather contrasting conclusions for the two parts of the ms. I think the topic is highly relevant, but the meta-analysis can only provide indirect evidence (as it did not focus on experiments where plants were simultaneously grown under different settings) and the own experiment was targeted but did not support the conclusion that glasshouses stand out as the artificial environment that cause distorted results. I am glad this reviewer is of a different opinion to the first in regard to the value of combining meta-analysis and experimental approach. I agree with what is said here in that the metaanalysis would have been ideally conducted on studies themselves comparing different experimental settings, however these studies are extremely rare, even if one were to include a variety of species rather than just one like I have done here, it probably would not provide the required statistical power. Within the new focus given to the manuscript, the two approaches now agree in that the mechanisms used by the plants seem to vary between settings, even if not in the same way in the experiment and meta-analysis. As explained now in the discussion, each approach provides a truncated answer to the research question, but the combination provides more certainty that there is a clear risk that plants are responding to salt stress with different acclimations across experimental settings. In my feeling, the ms can be made more valuable by (1) also including hydroponical systems as a category in the evaluation; (2) obtaining more observations by including durum wheat experiments and/or (3) possibly also by directly calculating and plotting dose-response curves rather than binning results. I think the author would agree that the point about the experiment would also be stronger if it was repeated a second time, as in good agricultural practice. But I can understand if that is a tough suggestion. My overall evaluation is mixed, and my suggestion is to give the author the option to resubmit an improved ms. The three main points listed here are addressed in detail in the responses to specific comments. Regarding the repetition of the experiment, I am in strong agreement. The results presented are actually the outcome of the second repetition of this experiment. I considered the results of the first iteration to not be reliable enough to be included here as the plants outdoors were not protected from the rain, which caused leaching of the salt from the outdoor pots. The main difference with the repetition presented here is that the outdoors pots showed the lowest effect of the salt stress, with moderate effect in the raised beds (still more than in the second repetition) and the most pronounced effect in the greenhouse (much higher than in the second repetition). In this first repetition, I already observed that the response pattern varied sharply between experimental settings, even if the exact outcome for each setting was different from the second repetition. This therefore still supports the overall findings of this study. Unfortunately, budget, space and time constraints did not allow me to repeat this experiment a third time. A. Meta-analysis 1. L141. Hydroponics are often used for assessing salinity effects. Although the author cites Tavanakki et al. as a source that hydroponics form an unrealistic situation, it would have been interesting to test the wider validity of that claim based on two specific experiments with a metaanalysis where results of hydroponics and pots are compared in growth chambers and glasshouses. Now we have to rely on observations in two papers, where a rather specific test was carried out. Is this not an interesting addition to this paper? I do agree that this would be very

interesting and I am definitely planning on researching that aspect in the future. However, given that this study is already complex I tried to limit the scope to keep the main message simple, which is in line with the recommendations of the editor. 2. L161. Comparing field sites that are up to 50 km away. How sure can you be that climate, and especially edaphic conditions are comparable, as well as management? Why not relying on experiments which are carried out on the same experimental field? Lack of degrees of freedom? An alternative would be to include durum wheat as well. They may be genotypically different, but there is also genotypic variation within the bread wheats that are the focus of this meta-analysis. This is a central issue with meta-analyses, in that the results are limited by the validity of the studies included, so there has to be some amount of trust in that the authors of those papers have ensured that the experimental locations have suitably comparable management, climatic and soil conditions. Many peer-reviewed articles I went through for this work have drawn conclusions on the effect of salinity stress based on a comparison of fields much further apart than 50 km (and were therefore eliminated). One can ask whether these results would have been published if completely invalidated by the differences between locations. Ideally, I would have preferred to only include experiments in the same field, as suggested here, but this very often meant that the salinity was created by artificial addition of salt rather than by naturally saline soil, which comes with its own limitation. In addition, restricting myself to fields in the same location would have reduced the amount of data available and therefore made results less reliable. The decision of a distance of 50 km ensured that the field data included naturally saline soils and optimised the amount of data for the analysis while excluding studies where differences in the conditions between control and stress were more likely. The decision to exclude durum wheat for this study was founded on the acknowledged fact that bread and durum wheat differ not only in their level of salt stress tolerance but also in the mechanisms involved in salt tolerance, with for example bread wheat displaying better homeostasis of sodium and potassium than durum wheat (Munns and Tester, 2008; Farooq et al., 2024). These differences could have confounded some of the responses to salt stress analysed here and potentially skewed the comparison between settings in case one species was more represented in some of the settings. 3. L184. “We only collected data where the salinity induced a decrease in either shoot dry weight or yield”. That seems an odd criterion, what is a-priori wrong with such experiments? There will always be random variation around an overall trend, but by omitting variation in one direction the overall trend will become biased and is not the overall trend anymore. This criterion is based on the generally accepted definition of stress “a ‘stressful environment’ is any environment that is less then optimal for plant growth” found in (Smith et al., 2009). 4. L184. “We only recorded data presented as amount per dry weight”. As far as I read the salinity literature, many authors like to express concentrations per unit fresh weight. I can also relate to dry weight, ideally you would have to express them in both ways to understand the changes. For chlorophyll, I would make an exception: Given that this related to photosynthesis, is it not more logical to express this per unit leaf area? It is entirely true that a sizable fraction of studies provides biomass results per amount of fresh mass. However, if looking at the effect of stress on the accumulation of a certain metabolite, you can only rely on concentration per dry mass given that both water content in the tissue and biomass are conflated in the measurement of fresh mass: an apparent increase in the concentration of a metabolite per unit of fresh mass could reflect that it is more concentrated in the tissue due to water loss caused by the stress rather than its accumulation. I agree that calculating chlorophyll concentration per unit leaf area is the most biologically meaningful metric, however it was very rarely used in the studies reviewed here. 5. L198. The categories are not

named fully consistently in the ms, is this not a good point to do so? (I would suggest CC, GH, OP and OB, to stress that outside pots and outside raised beds share the same outside). This was a mistake and has been corrected so that the acronyms are consistent throughout. 6. “Our analysis was based on the assumption that the effect of salt stress increased with the intensity of the stress”. I do not understand why that is a necessary assumption for the analysis? Isn’t this what the result of the meta-analysis could be, rather than an a-priori assumption? So what would you do with your own analysis, where you find salt-treated field plants also to produce more? Unfortunately, this criterion was necessary to eliminate (at least some of) the studies with faulty results that would skew the outcome of my analysis. I am not aware of any study deciphering the mechanisms of a non-monotone response of plant growth to the intensity of salt stress, and you would think that if this surprising result had been observed with certainty it would have been investigated further to understand the mechanism allowing the plant to grow better under more challenging conditions to inform crop improvement. This criterion does not apply to my own study as I only used a single level of salt. That said, I do agree there is a good chance that I have eliminated some valuable studies from the meta-analysis, but I judged that removing flawed studies was worth losing a small amount of reliable data. 7. L236-243. I am not sure whether the binning is the best solution. You mentioned that you were after dose-response curves. In principle, by binning you still could compare groups that differ in the exposure to salinity. Would it not be better to fit a line through all individual points? If you are worried about the effect of outliers, you could carry out a median regression. That also avoids having to impute values for the outliers, as is done now (I suppose?). Also, in the current analysis the control plants at salinity=0 are ignored. For an ANOVA I can see the point, because they all go to 1 because of the scaling used, but for the dose-response curve it is clear that this should go straight to 1, right? The justification for binning rather than fitting a continuous line is detailed in the “General answer on the data analysis approach and statistical methods used” at the end of this document. Regarding the control plants, there is no reason to include them, as the measurements presented are relative stress responses, and the control plants are not subjected to stress. This is generic practice in a meta-analysis, see for example (Cohen et al., 2021). Having a bin consisting of only control plants, all set at a value of 1 for each setting would bring to additional power to the analysis. 8. L434. “the effect … of stress is only significant for shoot weight”. So, is the conclusion here that there is no effect of salinity on root weight (Fig. 3b) and plant height (Fig. 3c)? Isn’t that unlikely? There are some explanations. First, there are only few observations in the 2 greenhouse groups (n=6), so this is not too many for a strong conclusion. Second, I think it is a consequence of the analysis, where control plants are not included (see point above). And lastly, maybe the salt stress gauge where salinity level is multiplied by the days of stress is not as linearly related to the stress. (Is it helpful to include in the appendix the graphs for salinity per se, next to those where salinity is multiplied by time? The author may be right, but I would like to see the result for myself). What is interesting is that for plants in the generative stage, plants in greenhouse are relatively less affected for their root dry weight than the other settings (Fig. 2b), whereas they are most strongly affected in Fig. 3b. No, that is not the conclusion from this figure. As stated above, the point of the analysis is not to detect a significance in the effect of the stress, but compare the different settings. In that process, in the original version of this manuscript we have also analysed whether the extent of the response is significantly affected by the level of the stress, however, this aspect has now been removed to better focus on the comparison between settings. I don’t believe the results of figure 2 and 3 can be compared as the stress levels are measured in different ways. 9. L458. This is an

interesting result, with sodium increasing so much stronger in greenhouse conditions! At the same time, could this graph also be used to prove that different stress levels do not affect [Na] in any of the other settings? The lines are basically horizontal, and that is even more unexpected to me than the steady increase for plants in the glasshouse. How can that be explained? For the other settings than the greenhouse, the response of the plants for sodium accumulation does seem to depend little on the stress level, but the plants did accumulate sodium (about 2-3 times more than in control conditions, as represented by the relative response). This could suggest than in these settings plants exclude salt uptake past a certain level of accumulation (which could be beneficial to osmotic adjustment) to avoid salt tissue toxicity, and they are able to do so regardless of the concentration of salt in the soil. This explanation is quite speculative though, and I am reluctant to include it in the discussion. B. Experimental study 1. L257. Much better than 50 km! Picture is informative, we should standard add pictures of our experiments to the papers! Please start with that habit and also add pictures of the glasshouse and growth chamber experiment. As suggested, I have added to supplementary figure S1 photos of the climate chamber and greenhouse experiments. 2. L268-275. Growth conditions are not clear to me. Table 2 in pdf format is a mess, but that maybe due to import problems by the journal's website? Anyway, it is mentioned in the text that photoperiod and day and night T were set in the growth chamber as they were in the glasshouse (the week before?), but in Suppl. Table S2 I only see mean, min and max 24 h data, no day and night T. Daylength is not provided at all. Moreover, it is not so interesting what the setpoint temperatures were in the growth chamber, but what actually has been realized is relevant, please provide those data in summarized form. Also, daily light integrals are missing, both for the field, the glasshouse and the growth chamber. Also unclear what soil medium was in the raised beds below 50 cm. Density in the raised beds was 220 plants/m2. What was it in the other settings, and if it was lower, could it be discussed whether that possibly affected the results? Daylength has not been provided because (1) it is not generally a parameter included in the description of environmental conditions given that it can be easily inferred from the location and dates of the experiment if one was interested in knowing these things and (2) this is not a parameter that varied between experiments. Unfortunately, the data necessary for the calculation of daily light integrals was not collected during these experiments so this data cannot be provided. It was not deemed necessary to measure as it is very rarely reported in abiotic stress field studies. I also did not record the actual temperature in the growth chamber, but the growth chamber has a display of measured temperature next to set a temperature and regular checks during the experiment have shown that measured and set temperatures were very similar for the whole time. The natural soil of the experimental garden was underneath the first 50 cm of the raised beds. This has been added to the relevant methods section. Density values are not very meaningful for the pot experiments given that most of the pots were not entirely surrounded by neighbouring plants, especially in the climate chamber and outdoors experiments where all pots were on the edge. In addition, density varied during the experiment as plants were sacrificed from some pots for shoot tissue collection. If one wanted an approximation of density, they could estimate it from the photos of the experiment provided in supplementary figure 1. Some discussion of the contribution of differences in density between settings has been added to the discussion. 3. L284-306. Great to use a mix of salts, but reference to a website for its composition is unsatisfactory, a website is too unstable. Please provide the details in the supplement. I am not a salinity expert, but 27 dS/m sounds like a very saline solution to me. That is more than half the strength of seawater! This of course depends on the volume of soil as well, but with 50 ml of this solution added for the

duration of the experiment (90-110 days), this adds up to 2 liters of salt solution. It was not mentioned what pot water capacity was (why not? Seems relevant to me), but if I assume it is 30% (v/v), a 3 liter pot ends up over the experiment with the same concentration as full seawater (uptake exempted). I know the author has written a viewpoint about making experiments not too simplistic (and I fully sympathize), but is this a stress that is realistic? I was not sure to which treatments the EC1:5 values refer to, the text is not fully clear here, but if they relate to the salinity treatment, how does it come that EC values are so much lower than what was added in total? Was that taken up by the plant? Or was there any attempt to regularly flush the salts in the pots? It is not mentioned. The composition of Instant Ocean has been added to the supplementary data (supplementary table S3) and mentioned in the methods section. The estimations of the salinity of the soil solution of this reviewer match my own, in that it would have been on average 35 g of Instant Ocean salt per litre of soil solution, which is just above the salinity of seawater. This is consistent with the EC1:5 values obtained, which are of an average just above 10 dS/m. As these EC1:5 values, they need to be multiplied to obtain classic EV values, i.e. 50 dS/m which is roughly the EC of seawater. There is therefore no inconsistency in these measurements. This is indeed not a realistic level, but the pot experiments were not meant to be realistic, only to be comparable to the more realistic raised bed experiment, where the soil EC values were much lower, and in line with what would be observed in natural saline fields: average EC1:5 of 3 dS/m over the first 30 cm of the soil, equivalent to 15 dS/m of classic EC measurement. This is indeed at the top of the range of field salinity reported in the meta-analysis part of the paper, but still within that range. I have amended the methods section to clarify that the pots were not flushed at any point. The text states that the EC1:5 measurements were obtained "at the end of the experiment at different depths for the salt treatments" and that "Similar results were obtained for the pots in all three settings". To make this clearer, as suggested, I have rephrased those sentences: ""at the end of the experiment at different depths for the pots that had been watered with the saline solution" and "Similar results were obtained for the pots in all the CC, GH and OP settings" 4. L304. "Chlorophyll extraction was modified from Sims & Gamon (2002)". How? One of the differences is that Sims & Gamon used frozen leaf material, whereas here the material was freeze dried. Is freeze drying and the subsequent storage at room T (?) not causing breakdown of part of the chlorophyll? There have been indeed some reports of chlorophyll degrading during storage after freeze drying, however, this mostly happens when samples are kept at high humidity levels (Östbring et al., 2020). The tissue samples in this study were stored in tightly shut centrifuge tubes placed in sealed plastic bags containing silica gel that was replaced when the colour changed to indicate it had absorbed humidity. It is therefore reasonable that the samples were kept at very low humidity and had low level of degradation. 5. L520. It is very helpful in these graphs if the y-axis starts at 0, not undefined as in Fig 5b, or some negative value as in the other panels. Please adjust the standard settings of R. Also, would it not be better to calculate the ratios on the median values rather than the mean ones? Third, are what you design as outliers included in calculating median values? In case the outliers are not included: why is that information neglected in a variable/parameter which anyway is designed to minimize the effect of outliers? Fourth, various relative values listed above the boxplots seem wrong, see e.g. the CC plants in Fig. 5b. Fifth, what should we think of the fact that in RB salinity increases yield? 1.The figure has been modified accordingly to this comment. 2. If this had been an independent experiment, it would have been a good idea to calculate the ratios based on the median, however here the ratios have been included to allow comparison with the meta-analysis, and in the meta-analysis the ratios were calculated

based on the average data provided from studies, so for the sake of keeping the data comparable between the two parts of my study. In addition, in the case of the raised beds, the average of yield is more likely to give an accurate representation of yield per surface area, as is usually measured in field conditions, than the median. 3.While outliers were removed in the meta-analysis, this was not done for the experimental data, all the data was included in the calculations of both means and medians. The outliers are only removed in the process of calculating the whiskers of the plot to represent the range of the date, but for nothing else. 4. Yes, the ratio value for CC in figure 5b is very obviously wrong, and this has been corrected. The values of the other ratios have been checked and are accurate 5.An explanation of this unexpected results has been included in the discussion, in the limitation section: "In our experiment, the salt stress unexpectedly led to positive, but nonsignificant, effects on yield and shoot growth in the raised beds. We suspect that this result was not due to the salt stress but to an unidentified pathogen that affected each of our four raised beds plots unequally. It was difficult to quantify the exact impact of this pathogen on yield, and while we aimed to exclude plants visibly infected from our measurements, it is possible that our salt stress plots were less affected by this pathogen, confounding the effect of high salinity. Without this pathogen, the difference in the effect of the stress between the raised beds and the other experimental settings may have been smaller, but we believe this issue does not invalidate the difference in acclimation strategy between experimental settings, in particular the accumulation of Na+." 6. Fig. 7a. Now I get confused: The RB+ treatment shows Na+ values close to 700 umol/g, which is 1.6% on a w/w basis. This is far more than plants in any of the other treatments, and yet, these are the plants that increase their yield in the saline treatment. At first sight, that does not seem consistent, how should we interpret this? The concentration of sodium found in the stressed RB is within the range of what has been found in other studies, for example (El-Hendawy et al., 2017) reported as high as 2500 µmol/g of DW at flowering stage in wheat leaves. I completely agree that this is a surprising result given the absence of deleterious effect of the salt stress on yield in this setting, but it still can be explained and I have included a section in the discussion for that purpose: "Another important inconsistency between the two approaches is for the accumulation of Na+ under field conditions: in our experiment, the salt stressed raised bed grown plants reached a 30-fold increase in shoot Na+ compared to the control, while in our meta-analysis we found that this increase was generally lower than three-fold. This is particularly intriguing given how little the shoot growth of our raised beds plants was affected by the salt stress. It could suggest that in the conditions of our raised beds the acclimation put in place by the plants was similar to that of halophytic plants, using a resource efficient form of osmotic adjustment (Hopmans et al., 2021)." C. Discussion 1. L622 & L675. The suggestion that there is higher transpiration in the glasshouse is an interesting one. Glasshouses at high latitude do not heat up as easily as those at lower latitude, but at lower latitude they are often cooled with ventilators, which would aggravate the transpiration even more. Could you find any support in your dataset for a difference in response between glasshouses with different location in the direction you expect? Or otherwise maybe in the amount of water you provided to your own plants outside as compared to those in the glasshouse? That was actually not what I meant to suggest (though it is indeed an interesting explanation), I was merely referring to heat's direct stressful effect. While what is suggested may bring some clues on whether the ventilation of greenhouses is an important element of differences between experimental settings, with the editor's request to keep the focus on the main point, I feel that adding a figure analysing the effect of latitude on greenhouse stress response to support a small element of explanation is not the best decision.

Soil moisture was kept at the same level in all settings in my experiment, so that is not the explanation. 2. L698. So, does that imply that salinity effects cannot be studied at higher latitudes? This is a very valid question. Research at higher latitude would be relevant to salinity stress encountered with a comparable climate, for example in coastal areas, if inland land is not affected by high salinity, but overall saline soils are much less of an issue at higher latitudes and therefore experiments conducted under those climates may have limited impact for those areas where this stress is a major contributor to the yield gap. Given that the section this comment is about has now been removed, this reflection has not been included in the manuscript. 3. After reading Tavakolli et al., I am not convinced that their experiment is sufficient to warrant such a claim. It would be wonderful if you could show similar results as they had in their experiment by adding hydroponical salinity experiments as an additional class in the meta-analysis. There are actually two papers by Tavakkoli at al. which together make quite a strong case for the limitations of hydroponics screening, in my opinion. See answer above about including hydroponics within this study. 4. L715-723. Wind stress can be strong and continuous in glasshouses at low latitudes equipped with large ventilators. (see above) This is a really relevant point, which I had not considered and has now been included in the discussion. 5. In the end, I do miss a discussion of how serious the differences between the settings, as observed in the meta-analysis, actually are. I mean, they were found to be statistically significant in a number of cases, but is a 20% relative difference in results also quantitatively large enough to make everybody worry. Or would it in the end still lead plant physiologists to the relevant mechanisms? And yes, pots outdoor seem intuitively to be a better option, but we also know that year-to-year variation in the field is huge. Where does that lead us? The new version of the manuscript now makes a much clearer case that there are clear disparities in the pattern of the salt stress response, which would mean indeed that the wrong mechanisms could be studied by physiologists (but not only) in controlled conditions. As already pointed out to the other reviewer, replicability under controlled conditions is not necessarily better than some variability under outdoors condition. It may mean more iterations of experiments are needed to establish patterns common between years, but this will bring much more reliable results than replicable greenhouse/climate chamber experiments. D. Other remarks - l175 “chlorophyll leaf/shoot proline”. Unclear what is meant. A comma was missing here it has now been added, and I have replaced the “/” with “or” for more clarity. - l201. “Where plants were stressed in several settings”. Not clear why they are excluded. If these “settings” relate to some or all of the categories CC, GH, OP or OB, I would say that that is the information you are actually after… What is meant here is that the same plants experienced conditions across different settings, e.g. they were moved from a greenhouse to outdoors within the course of the experiment, or they were initially grown - l222. Reference to sciplot? This was indeed missing and has been added. - L244. Test for normality are pretty insensitive at a low number of observations, but very sensitive at high n. Is that a worry in your case? In both the previous version or the revised one, normality was tested on the entire dataset (i.e. all settings and bins together) for each variable, which provided high enough numbers and quite comparable between the different variables that I don’t think this is a concern. - L257. Within 15 m, great! Much better than 50 km. Good to hear. - L300. How can 14 cm be the bottom of the pot? From L268 I understand the pots were 20 cm tall? This is a good point. The sampling of the soil for EC measurements was done after the pots had dried, the soil substrate had shrunk and the level of the soil had considerably lowered from the top of the pot. This has been corrected to reflect that this was originally the bottom two cm of the pot. - L310. GP -> OP? This has been corrected. - L327. Does

“shoot biomass” includes the generative biomass? Yes, this has now been added to the text. - L357-358. Given that non-parametric tests generally are less sensitive than ANOVA’s, it would be good if the reader could know which test was carried out when. Plant weights of individual plants often do show a log-normal distribution. No attempt was made to first logtransform the data? This may have strong consequences for yes/no finding an interaction significant… In the revised process for data analysis, we also tested normality of log-transformed data. Figure legend now includes the detail of the statistical tests performed. - L376. “Out-GH” not clear to me what contrast this is.. This has been corrected. - L389. Not specified what the error bars are. Why does the x-axis not start at 0? How can it be that the three groups have exactly the same x-value? One would not expect that…. If you would not want to plot all individual points, should there not at least be some error bar in the x-direction? Information on the error bars has been added. Because figures 1 to 4 (of the original version) are presenting relative responses (due to requirements of normalisation between studies, which is the standard of meta-analyses) rather than absolute values of measurements, the meaningful value on the y axis is 1, which represents the absence of effect of the stress and is as a point of reference on all of the meta-analysis plots. It is indeed not the case that all settings have the same salt intensity average. I simplified the figures by having a single value (the overall average for all settings) but I agree that this is misleading, I have therefore plotted the data again with the actual average stress intensity for each individual setting. You will notice that in most cases they are very close. As stated to a comment to the other reviewer, including horizontal error bars would be relevant if the analysis included comparing the different bins with each other, but this is not the case anymore. I preferred to provide information on the size and content of the bins in the tables included in the figures as I found that horizontal error bars impaired the legibility of the results as the different lines are often close to each other. - L411 “after the vegetative stage”-> “in the generative stage” ? This has been corrected accordingly. - L417. No plants “after the vegetative stage” in the growth chamber There were very few studies that went into the generative stage, not enough that they could be included in the analysis. - L444. Why are the lines for plants in pots in greenhouses dashed in Fig. 2, but continuous in Fig. 3? I have now homogenised the line format across the different figures. - L508. “Field” is maybe not the right word for these raised beds, with an artificial substrate? I agree, and this has been corrected. That said the substrate cannot be considered artificial when it is soil from a local field. - L541. It is helpful for the reader if all ratios in the figs 6c&d are listed in the same number of digits.Start y-axes at 0. This has all been changed accordingly. - L599. Are the presented graphs not a bit rudimentary to serve as dose-response curves? This section has been removed from the new version of the manuscript. - L787. Has the author considered to include the references for the meta-analysis in the reference list, as a small token of appreciation for all the authors of the original data? This is generally not done when the number of articles included in the meta-analysis reaches several hundred. In my case this would increase the number of references to over 300 and I am not sure whether the editor would allow it, but maybe they can let me know if they would prefer this is done. General answer on the data analysis approach and statistical methods used I very much appreciate the suggestions made by the reviewers, as they have identified some issues in my approach and their comments have guided me in designing improvements in the statistical analysis. I acknowledge that the statistical analysis applied to unbalanced designs was not suitable, so this has been entirely revised. To handle the lack of balance in sample numbers between settings, I applied a permutation based t- test (when t-test assumptions were satisfied), which provides more reliable results for unbalanced designs (Keselman et al., 2002) and a Mann-Whitney U test test otherwise. Both

reviewers had concerns about the approach undertaken in this study for the metaanalysis that consisted in using bins to construct the dose-response curves rather than inferring continuous dose-response curves. As mentioned in the methods section, it is essential for the quality of a meta-analysis to include a step to remove outliers from the analysis. Reviewer 2 suggested using a median regression to minimise the impact of outliers. I have thoroughly investigated this option, testing many different approaches using the quantref R package. I found that only linear median regression could be applied to my dataset as the different options available for non-linear regression were not suited for the main goal of my analysis, the comparison between groups (the four experimental settings), especially when the shape of the dose-response curve would differ between settings. In that case, I would have needed to apply different types of regression between settings, and this would have made statistical comparisons impossible. In addition, most of the non-linear regression options available for median regression involved a priori assumptions about the shape of the dose-response curve, but because of varying ranges of doses available for stress levels across the different settings, inferring the curve in the same way was often not possible. I was therefore left with the only option of linear median regression. As observed for yield, root dry weight at generative stage, shoot sodium or chlorophyll, the dose-response curves obtained using the bins are not linear therefore assuming a linear response means potentially skewing dose-response curves that would otherwise show differences between settings. You can see an example of the difference between the two methods in the plots below.
